# Supplementary material for: Abnormal dopamine receptor signaling allows selective therapeutic targeting of neoplastic progenitors in AML patients
Source: Cell Rep Med. 2021 Feb 16;2(2):100202. doi: 10.1016/j.xcrm.2021.100202 (PMC7897800; doi:10.1016/j.xcrm.2021.100202)
Supplement: Document S1. Figures S1–S5 and Tables S1–S5 [file mmc1.pdf]

Cell Reports Medicine, Volume *2*

Supplemental Information

Abnormal dopamine receptor signaling allows selective therapeutic targeting of neoplastic progenitors in AML patients

Lili Aslostovar, Allison L. Boyd, Yannick D. Benoit, Justin Di Lu, Juan Luis Garcia Rodriguez, Mio Nakanishi, Deanna P. Porras, Jennifer C. Reid, Ryan R. Mitchell, Brian Leber, Anargyros Xenocostas, Ronan Foley, and Mickie Bhatia

Supplemental Figure 1

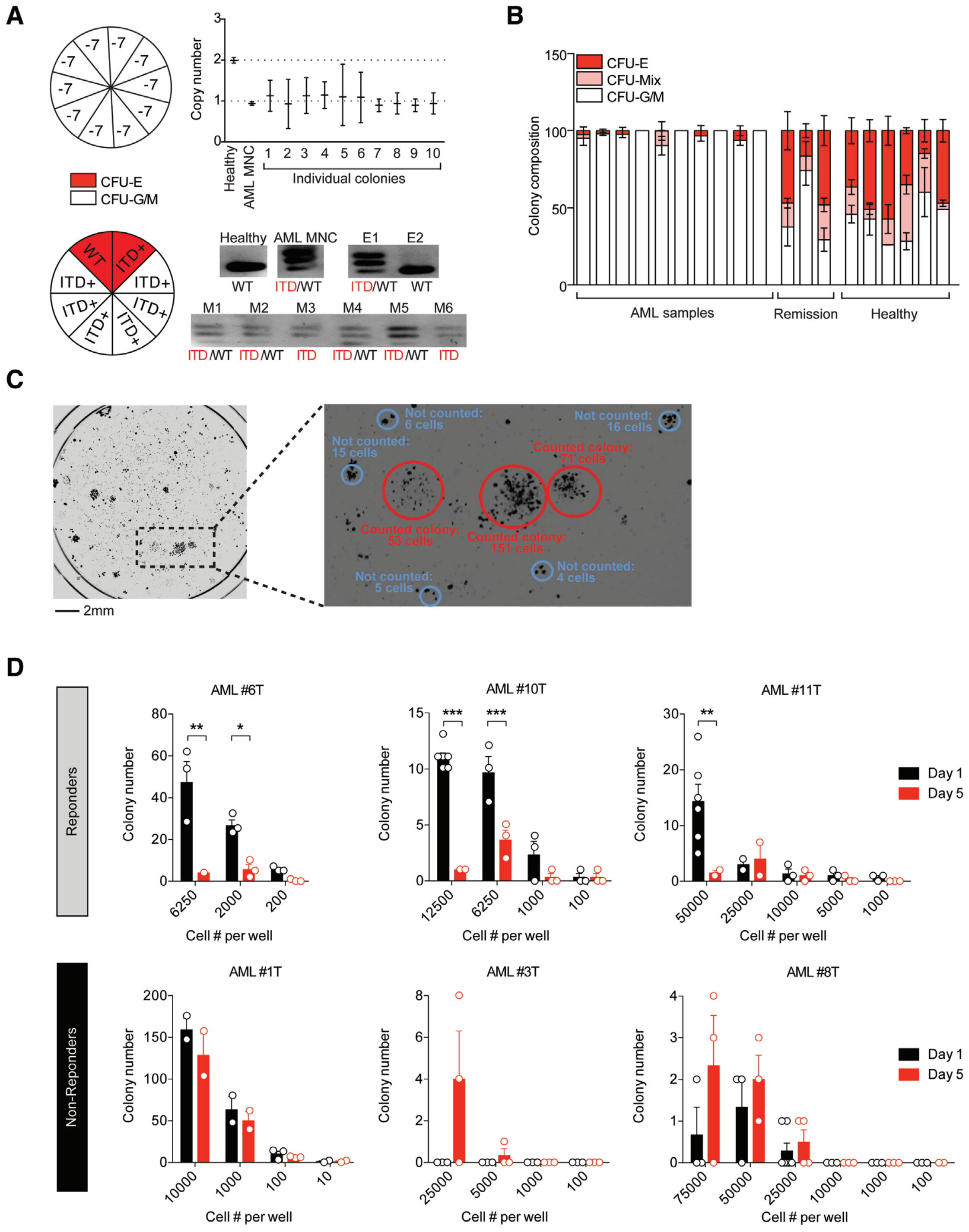

Figure S1. Characterization of AML patient samples for progenitor assays. Related to Figure 1. (A) Quantification of AML-related aberrations in granulocytic/monocytic ("G/M") versus erythroid ("E") colonies from two independent AML patients. Chromosome 7 deletion ("-7") was analyzed using droplet digital PCR system in 10 independent colonies by copy number variation analysis. Error bars indicate Poisson-based 95% confidence interval. FLT3-ITD mutation was tested using qRT-PCR analysis. Patient MNCs were used as a positive control and healthy donor samples were used as a negative control. (B) Based on Figure S1A, AML patient samples with over 90% enrichment for "G/M" colonies were prioritized for progenitor analyses, in contrast to diverse colony subtypes derived from healthy donor samples including CB and MPB and 3 remission cases of AML. (C) A representative whole well image shows contrast-adjusted calcein fluorescence of CFU colonies. The higher magnification image provides an example of our criteria used to score individual colonies for all experiments, based on a minimum requirement of 40 cells. Red circles indicate colonies that were counted, while blue circles indicate clusters that were not counted. (D) Candidate trial patient samples from either response group were interrogated for progenitor content at baseline (Day 1) and after clinical exposure to TDZ (Day 5) using limiting dilution analysis (LDA). *p<0.05, **p<0.01, ***p<0.001 (two-way ANOVA).

Supplemental Figure 2

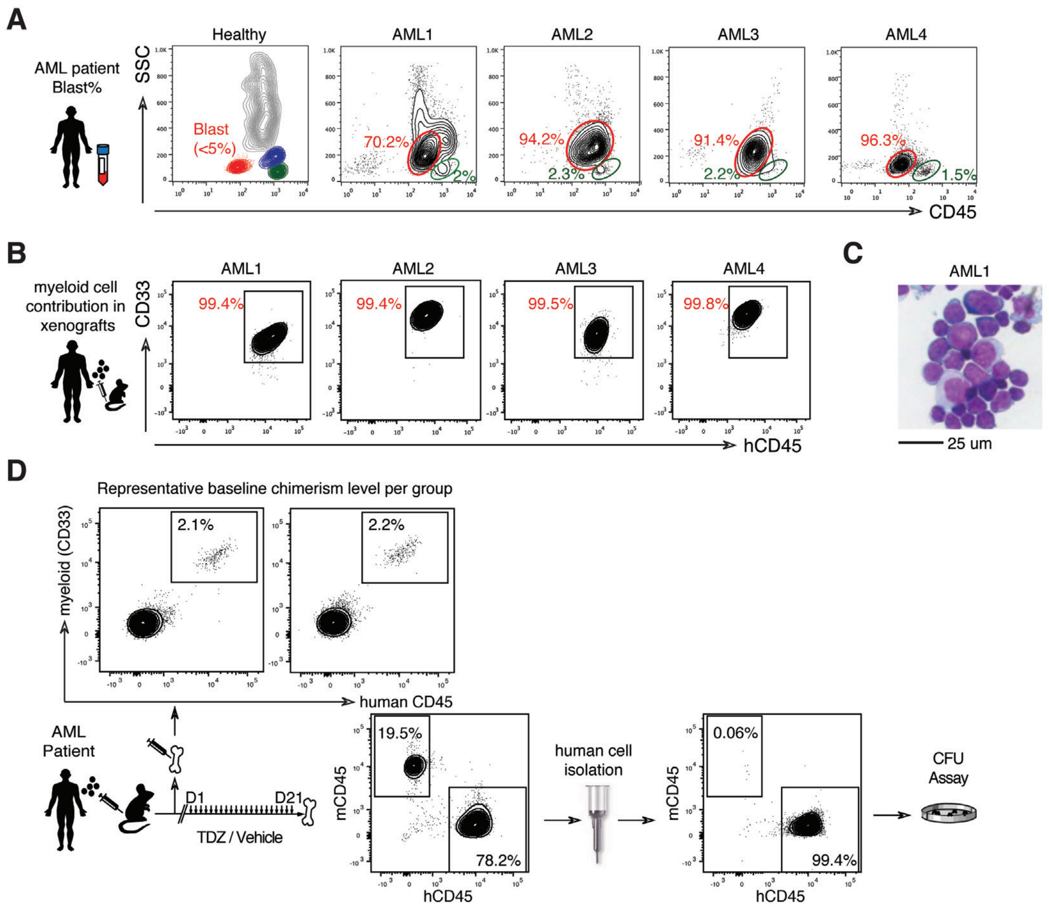

Figure S2. Characterization of AML patient samples for xenograft assays. Related to Figure 1. (A) FACS plots illustrate % leukemic blast (red gate) in AML patient samples. Green, blue and grey gates represent lymphocyte, monocyte and granulocytes, respectively. (B) AMLs 1-4 generate exclusively myeloid grafts in vivo. (C) Cytospin preparations showing blast morphology among human AML cells isolated from xenograft BM (AML 1). Original image capture had limited resolution and highest resolution is shown. (D) Recipient mice were assigned to TDZ versus vehicle control groups based on chimerism levels as quantified by BM aspirate sampling at approximately 3 weeks post-transplant, prior to the start of TDZ treatment. Representative FACS plot depicts the purity of isolated human leukemia cells prior to analysis of progenitor activity at the experimental endpoint.

Supplemental Figure 3

A

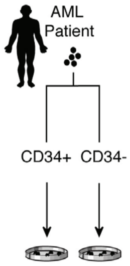

	CD34+		CD34-			
	Cell dose 1	CFU⁺ wells from total tested	Cell dose 1	CFU⁺ wells from total tested	Cell dose 2	CFU⁺ wells from total tested
AML 1	25k	3 of 4	5k	0 of 4	25k	0 of 4
AML 2	9k	3 of 3	9k	0 of 4	25k	0 of 4
AML 3	30k	4 of 5	30k	0 of 2		
AML 5	15k	1 of 4	15k	0 of 4		
AML 6	5k	3 of 3	5k	0 of 3	25k	0 of 3
AML 7	5k	3 of 3	5k	0 of 4		
AML 9	20k	1 of 4	20k	4 of 4		
AML 10	25k	4 of 4	25k	0 of 4		

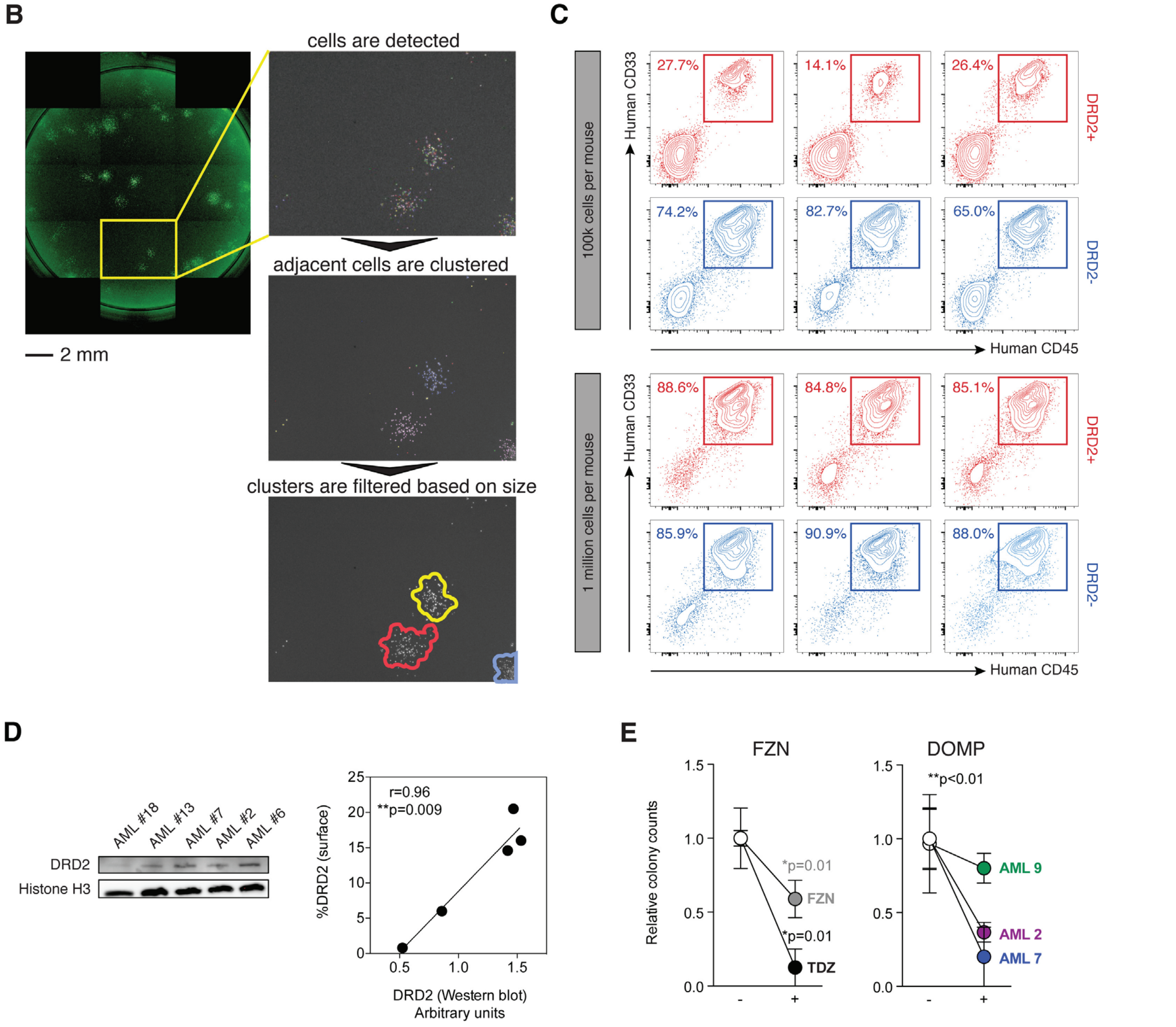

Figure S3. DRD2 signaling is functionally relevant to AML disease. Related to Figure 3. (A) CD34+ and CD34- subsets were FACS purified from AML patient MNCs for quantification of progenitor frequencies at various cells doses. (B) Identification of individual clusters and enumeration of cells within clusters as a measure of progenitor proliferative capacity using custom scripts. Original image capture had limited resolution and highest resolution is shown. (C) Human leukemic chimerism in mice transplanted with DRD2+ or DRD2- human AML cells. Note that plots from two of the mice at the 1 million cell dose are also shown in Figure 3E. (D) Western blot of DRD2 and Histone H3 (loading control) in 5 different primary patient samples (left). Correlation plot of cell surface DRD2 protein levels measured by flow cytometry vs. total DRD2 protein levels measured by Western blot. *p=0.009 (Pearson's correlation). Note that Western blot for patient AML 9 is also shown in Figure 3F. (E) Comparison of leukemic progenitor suppression after treatment with TDZ, fluphenazine hydrochloride ("FZN") and domperidone ("Domp") versus DMSO ("-"). FZN and TDZ treatments were performed using cells from patient AML 9. *n*≥ 3 CFU wells per condition. *p=0.01 (unpaired t-test), **p<0.01 (two-way ANOVA).

Supplemental Figure 4

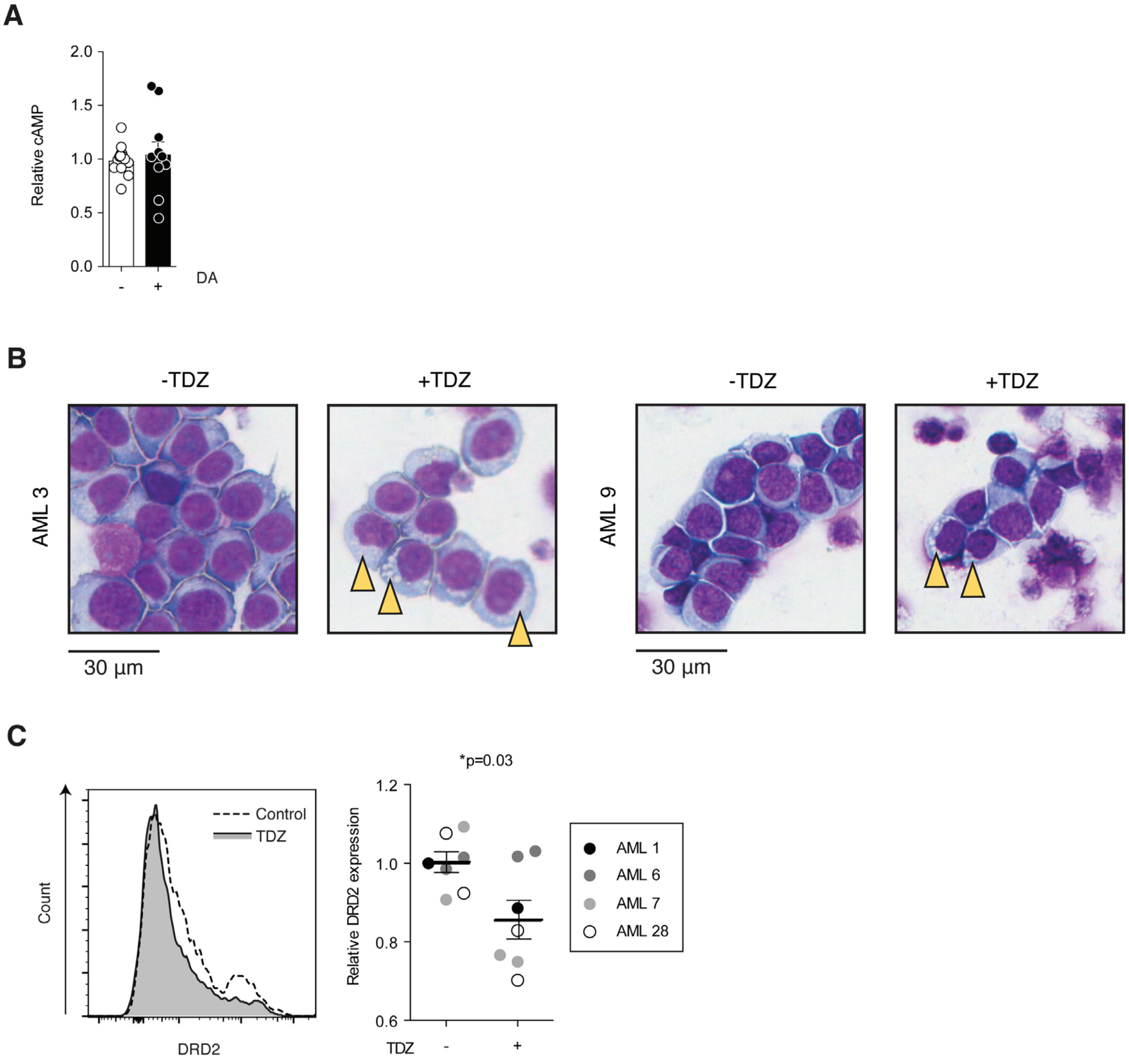

Figure S4. DRD2 signaling promotes cellular maturation and loss of self-renewal. Related to Figure 4. (A) cAMP levels in response to DA (100 nM) versus DMSO control, in AML cell line OCI-AML3. Data represent normalized values from *N*=5 independent experiments. (B) Cytospin preparations of AML after exposure to TDZ or vehicle control (DMSO). Yellow arrowheads indicate evidence of hematopoietic maturation (increased cytoplasmic vacuolization). Original image capture had limited resolution and highest resolution is shown. (C) FACS plot showing expression of DRD2 after in vitro exposure to TDZ or DMSO control (left). DRD2 frequencies were quantified for AMLs 1, 6, 7, and 28 (right). *p=0.03 (unpaired t-test).

Supplemental Figure 5

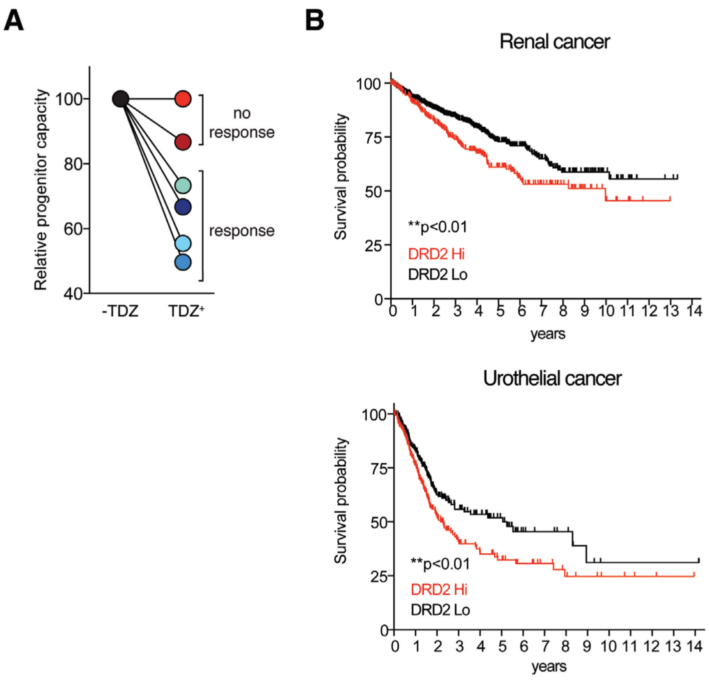

Figure S5. DRD2 is aberrantly expressed in tumors beyond AML. Related to Figure 5. (A) Trial patient samples obtained at baseline were exposed to TDZ+ versus DMSO control ("-TDZ"), followed by analysis of progenitor capacity. Patients included #1T, #8T for non-responders, and #2T, #6T, #10T, and #11T for responders. Data are normalized to DMSO control. (B) Survival analysis in cancer patients expressing high levels of DRD2 "Hi" versus low "Lo". Kaplan-Meier curves were generated using TCGA RNA-seq data with survival annotations provided by the pathology atlas. Log-rank test was used for statistical evaluation.

Table S1. Clinical details of AML patient samples. Related to Figure 1.

AML Sample ID	Disease stage	Cytogenetics	Molecular	Risk group*	%DRD2 in CD34+	%DRD1 in CD34+
1	Diagnosis	Normal	FLT3-ITD+, NPM1-	Intermediate-I	3.7	26.5
2	Diagnosis	NA	NA	NA	16.8	NA
3	Diagnosis	45, XX, del(5)(q22q33), -7[4]	NA	Adverse	NA	NA
4	Diagnosis	NA	NA	NA	NA	NA
5	Diagnosis	46, XY, 38 double minutes	NA	Intermediate-II	3.2	6.99
6	Relapse	45, XY, inv(3)(q21q26.2), -7[25]	NA	Adverse	8.1	5.1
7	Diagnosis	Normal	FLT3-ITD+, NPM1+	Intermediate-I	11.8	10.5
8	Diagnosis	Normal	FLT3-ITD+, NPM1-	Intermediate-I	2.2	15.2
9	Diagnosis	Normal	FLT3-ITD-, NPM1+	Favorable	1.3	26.5
10	Diagnosis	Normal	CBFß/MYH11+	Favorable	2.2	8.4
11	Diagnosis	Normal	FLT3-ITD+, NPM1-	Intermediate-I	3.2	NA
12	Diagnosis	Normal	NA	NA	0	1.2
13	Diagnosis	Normal	NA	NA	0.1	NA
14	Diagnosis	46,XX,inv(16)(p13q22)[25]	NA	Favorable	1.5	NA
15	Diagnosis	46,XX,add(4)(q31),t(16;16)(p13;q22)[25]	NA	Favorable	0.7	NA
16	Diagnosis	NA	NA	NA	1.4	NA
17	Diagnosis	47,XY,+13[24]/46,XY[1]	NA	Intermediate-II	0.8	NA
18	Diagnosis	46,XX,inv(16)(p13q22)[4]/ 47,idem,+22[21]	NA	Favorable	0	NA
19	Diagnosis	46,XX,t(8;21)(q22;q22)[25]	NA	Favorable	0.5	NA
20	Diagnosis	Normal	NA	NA	4.4	NA
21	Relapse	Normal	FLT3-ITD+, NPM1+	Intermediate-I	9.8	26.9
22	Diagnosis	NA	NA	NA	2.6	NA
23	Diagnosis	Normal	FLT3-ITD+, NPM1+	Intermediate-I	0.8	9.1
24	Diagnosis	Normal	FLT3-ITD+, NPM1-	Intermediate-I	7.7	NA
25	Diagnosis	46~47, XY, +9[3]/46, XY, del(15)(q11.2q15)[3]/46, XY, del(16)(q22)[2]/46, XY[6]	NA	Adverse	5.6	12.4
26	Relapse	Normal	FLT3-ITD+, NPM1+	Intermediate-I	7.5	NA
27	Relapse	42~46,idem,del(3)(p22p24), der(3)inv(3)(p21q21) del(3)(q21),del(7)(q32),add(18)(q21), add(20)(p12)	NA	Adverse	NA	NA
28	Diagnosis	NA	NA	NA	NA	NA

*European LeukemiaNet (ELN) criteria (Dohner et al., 2010)

Table S2. Gene Set Enrichment Analysis of upregulated pathways in control- vs. TDZ- exposed AML cells. Related to Figure 1.

Tissue	NAME	# of genes	NES	FDR q-val
Breast	SOTIRIOU_BREAST_CANCER_GRADE_1_VS_3_UP	138	-2.39	0.0000
	GINESTIER_BREAST_CANCER_ZNF217_AMPLIFIED_UP	65	-2.33	0.0000
	BERTUCCI_MEDULLARY_VS_DUCTAL_BREAST_CANCER_UP	188	-1.96	0.0019
	FARMER_BREAST_CANCER_CLUSTER_6	16	-1.92	0.0028
	JAZAERI_BREAST_CANCER_BRCA1_VS_BRCA2_UP	46	-1.90	0.0034
	POOLA_INVASIVE_BREAST_CANCER_UP	266	-1.87	0.0044
Lung	LOCKWOOD_AMPLIFIED_IN_LUNG_CANCER	198	-2.24	0.0000
	SHEDDEN_LUNG_CANCER_POOR_SURVIVAL_A6	425	-2.22	0.0001
	SWEET_LUNG_CANCER_KRAS_UP	456	-2.05	0.0007
	STEARMAN_LUNG_CANCER_EARLY_VS_LATE_UP	110	-1.91	0.0031
	KEGG_NON_SMALL_CELL_LUNG_CANCER	52	-1.66	0.0210
Blood	ROSS_LEUKEMIA_WITH_MLL_FUSIONS	68	-2.46	0.0000
	CASORELLI_ACUTE_PROMYELOCYTIC_LEUKEMIA_UP	162	-2.62	0.0000
	KEGG_CHRONIC_MYELOID_LEUKEMIA	69	-1.98	0.0016
	KEGG_ACUTE_MYELOID_LEUKEMIA	54	-1.93	0.0026
	PEPPER_CHRONIC_LYMPHOCYTIC_LEUKEMIA_UP	32	-1.81	0.0070
Liver	BOYAULT_LIVER_CANCER_SUBCLASS_G3_UP	178	-2.32	0.0000
	HOSHIDA_LIVER_CANCER_SUBCLASS_S1	220	-2.16	0.0002
	SAKAI_CHRONIC_HEPATITIS_VS_LIVER_CANCER_UP	76	-2.33	0.0000
Pancreatic, gastric and colon	GRUETZMANN_PANCREATIC_CANCER_UP	339	-1.94	0.0023
	KEGG_PANCREATIC_CANCER	67	-1.61	0.0295
	VECCHI_GASTRIC_CANCER_EARLY_UP	379	-1.91	0.0031
	AUNG_GASTRIC_CANCER	49	-1.65	0.0223
	GRADE_COLON_AND_RECTAL_CANCER_UP	258	-2.09	0.0005
Bladder	OSMAN_BLADDER_CANCER_UP	378	-2.91	0.0000
	LINDGREN_BLADDER_CANCER_CLUSTER_3_UP	305	-2.20	0.0001
Cervical	ROSTY_CERVICAL_CANCER_PROLIFERATION_CLUSTER	130	-2.51	0.0000
	OUELLET_OVARIAN_CANCER_INVASIVE_VS_LMP_UP	112	-2.38	0.0000
Other	WONG_EMBRYONIC_STEM_CELL_CORE	307	-2.17	0.0002
	WHITEFORD_PEDIATRIC_CANCER_MARKERS	107	-2.11	0.0003
	RAMALHO_STEMNESS_UP	193	-2.10	0.0004
	LIU_COMMON_CANCER_GENES	68	-1.77	0.0095
	LUI_THYROID_CANCER_CLUSTER_3	23	-1.66	0.0212
	KEGG_PROSTATE_CANCER	83	-1.52	0.0510

Table S3. KEGG acute myeloid leukemia gene list. Related to Figure 1.

Gene name	Gene description	Gene category/ family
AKT1	AKT Serine/Threonine Kinase 1	Protein coding/ Oncogene/ Protein kinase
AKT2	AKT Serine/Threonine Kinase 2	Protein coding/ Oncogene/ Protein kinase
AKT3	AKT Serine/Threonine Kinase 3	Protein coding/ Protein kinase
ARAF	A-Raf Proto-Oncogene, Serine/Threonine Kinase	Protein coding/ Protein kinase
BAD	BCL2 Associated Agonist Of Cell Death	Protein coding
BRAF	B-Raf Proto-Oncogene, Serine/Threonine Kinase	Protein coding/ Oncogene/ Protein kinase
CCNA1	Cyclin A1	Protein coding
CCND1	Cyclin D1	Protein coding/ Oncogene
CEBPA	CCAAT Enhancer Binding Protein Alpha	Protein coding/ Transcription factor/ Oncogene
CHUK	Component Of Inhibitor Of Nuclear Factor Kappa B Kinase Complex	Protein coding/ Protein kinase
EIF4EBP1	Eukaryotic Translation Initiation Factor 4E Binding Protein 1	Protein coding
FLT3	Fms Related Tyrosine Kinase 3	Protein coding/ Oncogene/ Protein kinase
GRB2	Growth Factor Receptor Bound Protein 2	Protein coding
HRAS	HRas Proto-Oncogene, GTPase	Protein coding/ Oncogene
IKBKB	Inhibitor Of Nuclear Factor Kappa B Kinase Subunit Beta	Protein coding/ Protein kinase
IKBKG	Inhibitor Of Nuclear Factor Kappa B Kinase Regulatory Subunit Gamma	Protein coding
JUP	Junction Plakoglobin	Protein coding
KIT	KIT Proto-Oncogene, Receptor Tyrosine Kinase	Protein coding/ Oncogene/ Protein kinase
KRAS	KRAS Proto-Oncogene, GTPase	Protein coding/ Oncogene
LEF1	Lymphoid Enhancer Binding Factor 1	Protein coding/ Transcription factor
LOC652346		
LOC652671		
LOC652799		
MAP2K1	Mitogen-Activated Protein Kinase Kinase 1	Protein coding/ Protein kinase
MAP2K2	Mitogen-Activated Protein Kinase Kinase 2	Protein coding/ Protein kinase
MAPK1	Mitogen-Activated Protein Kinase 1	Protein coding/ Protein kinase
MAPK3	Mitogen-Activated Protein Kinase 3	Protein coding/ Protein kinase
MTOR	Mechanistic Target Of Rapamycin Kinase	Protein coding/ Protein kinase
MYC	MYC Proto-Oncogene, BHLH Transcription Factor	Protein coding/ Transcription factor/ Oncogene
NFKB1	Nuclear Factor Kappa B Subunit 1	Protein coding/ Transcription factor
NRAS	NRAS Proto-Oncogene, GTPase	Protein coding/ Oncogene
PIK3CA	Phosphatidylinositol-4,5-Bisphosphate 3-Kinase Catalytic Subunit Alpha	Protein coding/ Oncogene
PIK3CB	Phosphatidylinositol-4,5-Bisphosphate 3-Kinase Catalytic Subunit Beta	Protein coding
PIK3CD	Phosphatidylinositol-4,5-Bisphosphate 3-Kinase Catalytic Subunit Delta	Protein coding
PIK3CG	Phosphatidylinositol-4,5-Bisphosphate 3-Kinase Catalytic Subunit Gamma	Protein coding
PIK3R1	Phosphoinositide-3-Kinase Regulatory Subunit 1	Protein coding/ Tumor suppressor
PIK3R2	Phosphoinositide-3-Kinase Regulatory Subunit 2	Protein coding
PIK3R3	Phosphoinositide-3-Kinase Regulatory Subunit 3	Protein coding
PIK3R5	Phosphoinositide-3-Kinase Regulatory Subunit 5	Protein coding
PIM1	Pim-1 Proto-Oncogene, Serine/Threonine Kinase	Protein coding/ Oncogene/ Protein kinase
PIM2	Pim-2 Proto-Oncogene, Serine/Threonine Kinase	Protein coding/ Protein kinase

Gene name	Gene description	Gene category/ family
PML	Promyelocytic Leukemia	Protein coding/ Transcription factor/ Oncogene
PPARD	Peroxisome Proliferator Activated Receptor Delta	Protein coding/ Transcription factor
RAF1	Raf-1 Proto-Oncogene, Serine/Threonine Kinase	Protein coding/ Oncogene/ Protein kinase
RARA	Retinoic Acid Receptor Alpha	Protein coding/ Transcription factor/ Oncogene
RELA	RELA Proto-Oncogene, NF-KB Subunit	Protein coding/ Transcription factor
RPS6KB1	Ribosomal Protein S6 Kinase B1	Protein coding/ Protein kinase
RPS6KB2	Ribosomal Protein S6 Kinase B2	Protein coding/ Protein kinase
RUNX1	Runt Related Transcription Factor 1	Protein coding/ Transcription factor/ Oncogene
RUNX1T1	RUNX1 Translocation Partner 1	Protein coding/ Transcription factor/ Oncogene
SOS1	SOS Ras/Rac Guanine Nucleotide Exchange Factor 1	Protein coding
SOS2	SOS Ras/Rho Guanine Nucleotide Exchange Factor 2	Protein coding
SPI1	Spi-1 Proto-Oncogene	Protein coding/ Transcription factor
STAT3	Signal Transducer And Activator Of Transcription 3	Protein coding/ Transcription factor
STAT5A	Signal Transducer And Activator Of Transcription 5A	Protein coding/ Transcription factor
STAT5B	Signal Transducer And Activator Of Transcription 5B	Protein coding/ Transcription factor
TCF7	Transcription Factor 7	Protein coding/ Transcription factor
TCF7L1	Transcription Factor 7 Like 1	Protein coding/ Transcription factor
TCF7L2	Transcription Factor 7 Like 2	Protein coding/ Transcription factor
ZBTB16	Zinc Finger And BTB Domain Containing 16	Protein coding/ Transcription factor/ Oncogene

Table S4. Progenitor CFU assays after *in vivo* exposure to TDZ. Related to Figure 1.

Patient sample	Cell # seeded per well	Colony count DMSO	Colony count TDZ	Average colony count (DMSO)	Relative colony count DMSO	Relative colony count TDZ
AML #1	100k	79	49	74.7	1.06	0.66
		69	49		0.92	0.66
		76			1.02	
	75k	61	29	54.0	1.13	0.54
		61	34		1.13	0.63
		40			0.74	
AML #2	25k	39	3	26.5	1.47	0.11
		43	2		1.62	0.08
		24	0		0.91	0.00
		18			0.68	
		20			0.75	
		15			0.57	
AML #3	25k	6	3	6.3	0.95	0.47
		5	4		0.79	0.63
		8	4		1.26	0.63

Table S5. TDZ suppresses leukemic progenitors exclusively in DRD2+ AML. Related to Figure 3.

Patient sample	Group	Colony count DMSO	Colony count TDZ	Average colony count (DMSO)	Relative colony count DMSO	Relative colony count TDZ
AML #1	DRD2+	47	9	32.0	1.47	0.28
		37	17		1.16	0.53
		16	5		0.50	0.16
		28	18		0.88	0.56
AML #5	DRD2+	89	30	82.8	1.08	0.36
		99	38		1.20	0.46
		59	49		0.71	0.59
		84			1.02	
AML #6	DRD2+	22	13	18.8	1.17	0.69
		13	8		0.69	0.43
		22	13		1.17	0.69
		18	5		0.96	0.27
AML #7	DRD2+	10	0	7.3	1.38	0.00
		3	2		0.41	0.28
		8	3		1.10	0.41
		8	1		1.10	0.14
AML #8	DRD2+	21	14	21.0	1.00	0.67
		16	19		0.76	0.90
		26	14		1.24	0.67
AML #9	DRD2+	2	0	2.0	1.00	0.00
		2	0		1.00	0.00
		1	0		0.50	0.00
		3	1		1.50	0.50
AML #10*	DRD2+	3	1	3.4	0.88	0.29
		5	1		1.47	0.29
		4	3		1.18	0.88
		3	1		0.88	0.29
		2	1		0.59	0.29
		3	0	1.4	2.14	0.00
		0	1		0.00	0.71
		1	1		0.71	0.71
		2	0		1.43	0.00
		1	2		0.71	1.43
AML #11	DRD2+	1	0	1.8	0.57	0.00
		1	1		0.57	0.57
		3	0		1.71	0.00
		2	0		1.14	0.00
AML #12	DRD2lo	9	36	15.3	0.59	2.36
		30	21		1.97	1.38
		11	2		0.72	0.13
		11	9		0.72	0.59
AML #13	DRD2lo	222	181	194.7	1.14	0.93
		204	181		1.05	0.93
		158	131		0.81	0.67

Patient sample	Group	Colony count DMSO	Colony count TDZ	Average colony count (DMSO)	Relative colony count DMSO	Relative colony count TDZ
Cord blood #1	Healthy	44	33	33.5	1.31	0.99
		29	41		0.87	1.22
		32	35		0.96	1.04
		29	30		0.87	0.90
Cord blood #2	Healthy	52	52	56.3	0.92	0.92
		57	52		1.01	0.92
		63	43		1.12	0.76
		53	39		0.94	0.69
Cord blood #3	Healthy	45	37	46.0	0.98	0.80
		50	40		1.09	0.87
		46	33		1.00	0.72
		43			0.93	

*Two independent experiments were performed with cells from AML #10
