# Supplementary material for: Abnormal dopamine receptor signaling allows selective therapeutic targeting of neoplastic progenitors in AML patients
Source: Cell Rep Med. 2021 Feb 16;2(2):100202. doi: 10.1016/j.xcrm.2021.100202 (PMC7897800; doi:10.1016/j.xcrm.2021.100202)
Supplement: Document S2. Article plus supplemental information [file mmc2.pdf]

Cell Reports
Medicine

Article

Abnormal dopamine receptor signaling allows selective therapeutic targeting of neoplastic progenitors in AML patients

Graphical Abstract

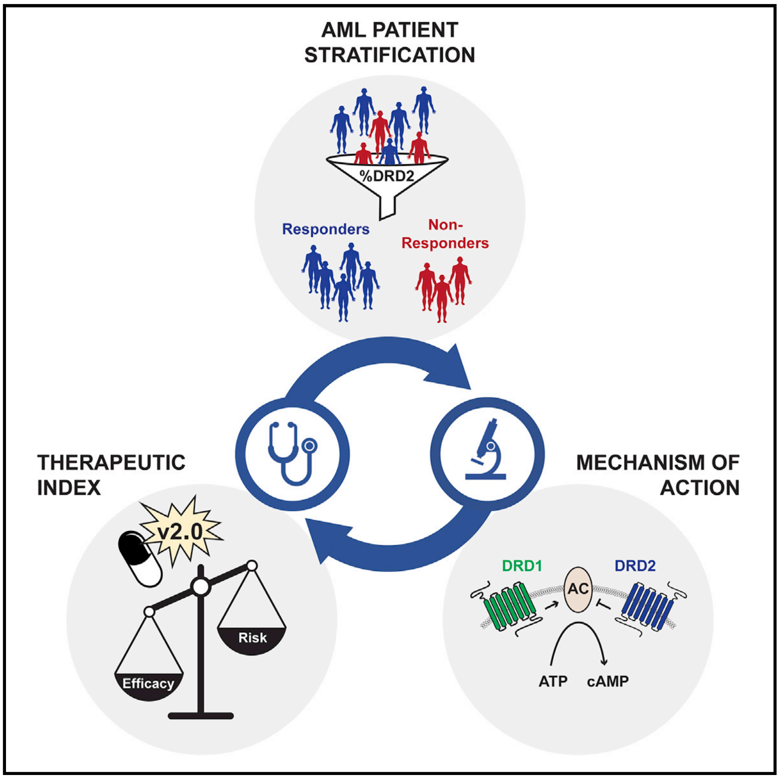

Authors

Lili Aslostovar, Allison L. Boyd, Yannick D. Benoit, ..., Anargyros Xenocostas, Ronan Foley, Mickie Bhatia

Correspondence

mbhatia@mcmaster.ca

In Brief

Aslostovar et al. establish reliable assays that predict clinical responsiveness to a dopamine receptor 2 (DRD2) antagonist using patient samples from a Phase I clinical study of acute myeloid leukemia. Using this platform, they identify biomarkers for patient stratification and develop an alternative drug formulation with an improved efficacy:risk ratio.

Highlights

- Leukemic progenitors are a critical cellular target of DRD2 antagonist TDZ
- DRD2 protein expression is a reliable biomarker of TDZ response
- DRD2 antagonism selectively triggers leukemic maturation programs via cyclic AMP
- An enantiomer of TDZ displays a superior efficacy:risk ratio relative to racemic TDZ

Aslostovar et al., 2021, Cell Reports Medicine 2, 100202
February 16, 2021 Crown Copyright © 2021
https://doi.org/10.1016/j.xcrm.2021.100202

CellPress

Cell Reports Medicine

CellPress
OPEN ACCESS

Article

Abnormal dopamine receptor signaling allows selective therapeutic targeting of neoplastic progenitors in AML patients

Lili Aslostovar,[1] Allison L. Boyd,[1] Yannick D. Benoit,[2] Justin Di Lu,[3] Juan Luis Garcia Rodriguez,[1] Mio Nakanishi,[1] Deanna P. Porras,[1,3] Jennifer C. Reid,[1,3] Ryan R. Mitchell,[1] Brian Leber,[4] Anargyros Xenocostas,[5] Ronan Foley,[6] and Mickie Bhatia[1,3,7,*]
[1]Stem Cell and Cancer Research Institute, McMaster University, Hamilton, ON, Canada
[2]Department of Cellular and Molecular Medicine, Ottawa University, Ottawa, ON, Canada
[3]Department of Biochemistry and Biomedical Sciences, McMaster University, Hamilton, ON, Canada
[4]Department of Medicine, McMaster University, Juravinski Hospital, Hamilton, ON, Canada
[5]Division of Hematology, Department of Medicine, University of Western Ontario, London Health Sciences Centre, London, ON, Canada
[6]Department of Pathology and Molecular Medicine, McMaster University, Juravinski Hospital, Hamilton, ON, Canada
[7]Lead contact
*Correspondence: mbhatia@mcmaster.ca
https://doi.org/10.1016/j.xcrm.2021.100202

SUMMARY

The aberrant expression of dopamine receptors (DRDs) in acute myeloid leukemia (AML) cells has encouraged the repurposing of DRD antagonists such as thioridazine (TDZ) as anti-leukemic agents. Here, we access patient cells from a Phase I dose escalation trial to resolve the cellular and molecular bases of response to TDZ, and we extend these findings to an additional independent cohort of AML patient samples tested preclinically. We reveal that in DRD2$^+$ AML patients, DRD signaling in leukemic progenitors provides leukemia-exclusive networks of sensitivity that spare healthy hematopoiesis. AML progenitor cell suppression can be increased by the isolation of the positive enantiomer from the racemic TDZ mixture (TDZ$^+$), and this is accompanied by reduced cardiac liability. Our study indicates that the development of DRD-directed therapies provides a targeting strategy for a subset of AML patients and potentially other cancers that acquire DRD expression upon transformation from healthy tissue.

INTRODUCTION

Acute myeloid leukemia (AML) is an aggressive hematological malignancy with poor prospects for survival. For the past 40 years, the standard of care has consisted of intensive chemotherapy, which is associated with substantial treatment-related morbidity.[1] Hematopoietic stem cell transplantation is offered to physically fit patients when suitable donors are available, but the majority of patients are not candidates for this preferred line of therapy due to the high toxicity burden involved.[2] The advancement of novel targeted therapies represents an important objective for the AML field, to improve selectivity toward leukemic blasts while sparing normal hematopoiesis.[3] The clinical development of new therapies is a challenging process in AML and requires iterative cycles of investigation that alternate between the laboratory and the clinic.[4] For example, careful evaluation of findings from early clinical trials has led to improved selectivity and potency profiles among second-generation fms-like tyrosine kinase 3 (FLT3) inhibitors[5] and has also revealed important mechanisms of acquired resistance to the guanosine analog ribavirin.[6] With similar goals in mind, here, we report on insights gained from the initial clinical investigation of a dopamine receptor-targeted therapy in AML patients, informed by follow-up laboratory analysis and experimentation.

Dopamine receptors (DRDs) are a class of G protein-coupled receptors (GPCR) and were originally identified in neural tissue as mediators of learning, memory, and regulation of sympathetic tone.[7,8] The 5 members of the DRD family are subdivided into D1-like (DRD1 and DRD5) or D2-like (DRD2–DRD4) receptors, each with disparate signaling and unique pharmacological properties[9,10] that have classically been targeted to treat psychiatric disorders.[11] More recently, the biological roles and therapeutic promise of DRDs were revisited when a well-established antagonist of DRD2, thioridazine (TDZ),[12] was identified in anti-cancer compound screens for both neural[13–15] and non-neural cancer cells.[14,16,17] TDZ also emerged as a front-runner to counteract universal oncogenic features of human cancer in an unbiased artificial intelligence-based analysis of nearly 170 compounds and 33 tumor types.[18]

Based on this promising preclinical evidence, the DRD2 antagonist TDZ was clinically evaluated in a recent Phase I study of AML patients with relapsed or refractory AML (NCT02096289). During a brief window of single-agent treatment with TDZ, leukemic blast cell counts were actively reduced in 8 of 11 patients, while 3 patients showed no evidence of disease alleviation.[19] Furthermore, patients classified as responders had significantly higher levels of cell surface DRD2 expression within leukemic blast

 Crown Copyright © 2021
This is an open access article under the CC BY license (http://creativecommons.org/licenses/by/4.0/).

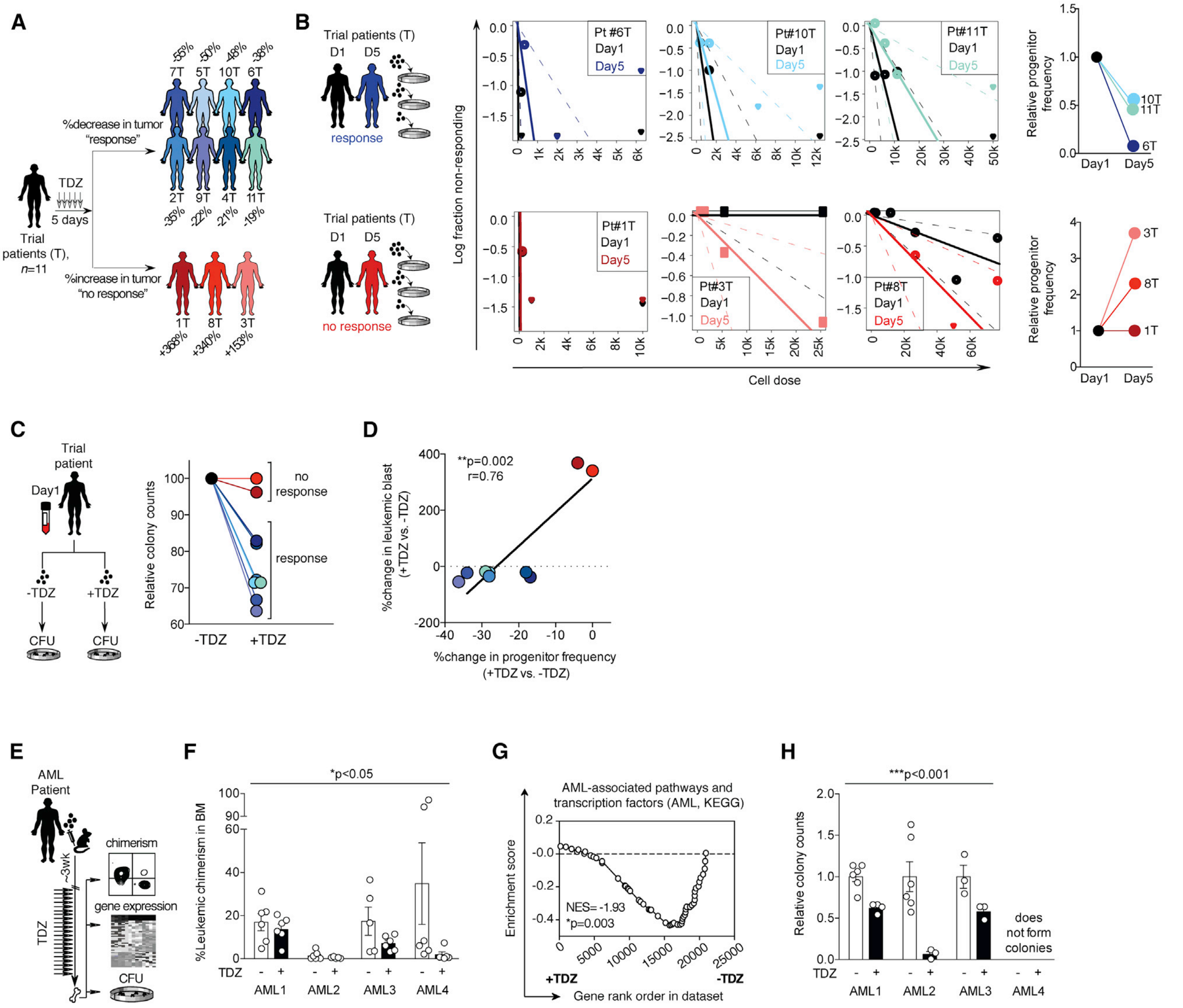

Figure 1. Leukemic progenitor assays replicate patterns of patient response to DRD2 antagonist TDZ

(A) Leukemic blast counts were monitored before and after treatment with TDZ as a monotherapy in 11 relapsed or refractory AML patients (NCT02096289). Percentage change in blasts in the peripheral blood on day 5 versus day 1 is reported after treatment with TDZ. Percentage change in BM blast content is reported for trial patient 2T and 9T in the absence of circulating blast values. Partial response and progressive disease patterns[19] are indicated as "response" and "no response" and are illustrated as gray versus black silhouettes, respectively.

(B) Candidate trial patient samples from either response group were interrogated for progenitor content at baseline (day 1) and after clinical exposure to TDZ (day 5) using limiting dilution analysis (LDA).[23] Leukemic progenitor frequency was estimated by LDA analysis and normalized to day 1. Baseline progenitor frequency of 1 in 75,000 cells was considered the progenitor frequency for trial patient 3T at day 1 since an absolute frequency was not achieved with the analysis of 75,000 cells for this patient. Dashed lines represent 95% confidence interval. Raw colony counts are shown in Figure S1D.

(C) Trial patient samples obtained at baseline were exposed to TDZ ("+TDZ") versus DMSO control ("−TDZ") for 24 h, followed by analysis of progenitor cell function in CFU assays. Data are normalized to DMSO control. Before normalization, the average DMSO control values were 79 and 2 colonies for trial patients 1T and 8T (non-responders) and 61, 28, 56, 2, 11, 28, and 14 colonies for trial patients 2T, 4T, 6T, 7T, 9T, 10T, and 11T, respectively (responders). Patients 3T and 5T were not included in this analysis due to a lack of detectable progenitor function.

(D) Correlation between percentage change in leukemic blast levels versus percentage change in progenitor capacity (demonstrated in C). Patients 3T and 5T were not included in this analysis due to a lack of detectable progenitor function.

(E) Schematic illustrating *in vivo* AML xenografts were treated with TDZ (22.5 mg/kg "+") or 30% captisol (vehicle control "−") *in vivo*, followed by analysis of leukemic chimerism levels (F), gene expression analysis (G), and progenitor CFU assays (H).

(F) Leukemic chimerism levels ($hCD45^+CD33^+$) after *in vivo* treatment with TDZ relative to vehicle control ("−"). Symbols represent individual recipient mice. *p = 0.05 (2-way factorial ANOVA). There was no significant interaction effect between patient sample and treatment group.

(G) Gene set enrichment analysis (GSEA) plot of a gene set representing cellular pathways associated with AML (Kyoto Encyclopedia of Genes and Genomes [KEGG]; Table S4), applied to transcription profiles from TDZ-treated versus vehicle control-treated AML xenografts derived from AMLs 1, 3, and 4.

(legend continued on next page)

populations.[19] While this study successfully identified a safe dose of TDZ for future use in AML patients,[19] dose escalation to achieve maximal efficacy remained a challenge due to adverse neurological and cardiac side effects associated with TDZ.[20,21] Encouraged by the preliminary signs of efficacy and the possibility to improve on these outcomes, we have returned to the laboratory bench to evaluate the mechanism of action and cellular basis of AML patient response versus non-responsive patients. Using AML patient samples from this trial together with the contextual clinical data, we characterize the cellular and molecular bases of TDZ therapy response, which can be exploited toward more refined, targeted, and safer DRD-based therapies. Our findings reveal the biological role of DRDs in the context of malignant hematopoiesis and provide foundational insights into DRD-directed therapies for use in AML as well as a broader range of non-neural cancers that co-opt the DRD pathway during healthy to cancerous transformation.

RESULTS

Leukemic progenitors are an important cellular target of DRD2 antagonist TDZ

A total of 13 patients were enrolled in a Phase I clinical trial to evaluate the safety of the DRD2 antagonist TDZ as a potential anti-leukemic therapy (NCT02096289).[19] The study was designed to include a 5-day lead-in period,[22] in which TDZ could be evaluated as a single agent in advance of the introduction of standard chemotherapy. Despite the brevity of this monotherapy window, a 19%–55% reduction of blast levels was observed in 8 of the 11 AML patients who completed the initial lead-in phase[19] (Figure 1A). Viable leukemic cells were collected from trial patients at the study's baseline and also after the 5-day TDZ treatment, providing an opportunity to interrogate therapy-related changes in disease composition on a patient-by-patient basis. Because TDZ was originally identified for its predicted ability to block leukemic self-renewal mechanisms,[16] we applied semisolid *in vitro* assays to quantify functional leukemic progenitors before and after clinical exposure to TDZ. Colonies were recognized to be leukemic in origin based on the presence of patient-specific aberrations (Figure S1A) and/or abnormal colony composition consisting of uniform myeloid colonies (Figures S1B and S1C). Quantitative limiting dilution analysis (LDA)[23] revealed an acute depletion of progenitor pools (1.9- to 23-fold), exclusively among patients who experienced some degree of clinical response (6T, 10T, and 11T; Figures 1B and S1D). In contrast, progenitor frequencies were unchanged in all 3 non-responding patients from the trial (1T, 3T, and 8T; Figures 1B and S1D).

We next designed an approach involving a liquid culture system, followed by the same AML progenitor assay readout to determine whether the observed progenitor responses in treated patients would have been predicted by exposing the same patients' naive cells to TDZ *in vitro* compared to treating the patients with TDZ themselves. This was performed using baseline therapy-naive AML samples obtained from each patient at the start of the trial. Following liquid culture in the presence of TDZ or DMSO (vehicle control), leukemic cells were then evaluated for progenitor content using the semisolid colony-forming assays. After only 24 h of culture, TDZ was able to reduce leukemic progenitor frequencies in 7 of 9 patient samples tested, matching the patient-specific patterns of response seen after clinical TDZ administration (Figures 1C and 1D). These findings indicate that the outcomes of *in vitro* TDZ treatment can predict leukemic progenitor responses seen in the clinic (Figures 1B–1D).

In parallel with the execution of the clinical trial, we structured a complementary xenograft study to mimic the design of the trial. This provided the benefit of evaluating *in vivo* treatment regimens in direct comparison to an internal vehicle control and also allowed the delivery of TDZ as a single agent for a full 3-week period. We established leukemic xenografts in NOD SCID mice via the intravenous transplantation of cells obtained from 4 distinct AML patients that were highly infiltrated with leukemic blasts (Figure S2A; Table S1). This required the use of patient samples collected independently from the trial because trial patient samples were limited either in cell number or engraftment capacity. All 4 of these samples exclusively generated pure myeloid xenografts (>99% $hCD45^{+}CD33^{+}$[24]; Figure S2B) with blast morphology (Figure S2C). In total, 46 individual recipient mice were treated with a clinically relevant dose of TDZ or vehicle control,[25] followed by cell purification of the human leukemic grafts (Figure S2D) for subsequent phenotypic and transcriptional analysis (Figures 1E–1G). *In vivo* TDZ treatment led to a 20%–95% reduction in leukemic disease levels compared to vehicle-treated controls (Figure 1F). This anti-leukemic effect was accompanied by a loss of gene expression signatures broadly associated with malignant transformation (Table S2), including a repression of hallmark cellular pathways specific to AML (Figure 1G; Table S3). To correspond with our analysis of progenitor content in TDZ-treated AML patients, we seeded human AML cells recovered from xenografts into the same *in vitro* progenitor assay. Leukemic progenitor cells within TDZ-treated xenografts were reduced in all cases, with the exception of AML#4, in which no progenitor activity was detected from xenografts, independent of TDZ administration (Figure 1H; Table S4). Overall, these results indicated that while a level of bulk leukemia cytoreduction was achieved in xenograft systems, TDZ exerts a strong anti-leukemic effect through the suppression of the progenitor compartment as seen in human patients. This positions the AML progenitor assay as a meaningful benchmark to judge the performance of new candidate molecules that target the DRD pathway.

DRD-directed targeting spares healthy hematopoiesis

Traditional chemotherapy treatment is non-selective and causes substantial damage to the healthy hematopoietic system,

(H) Human AML grafts were recovered from mouse BM and evaluated in progenitor CFU assays. Symbols represent individual CFU wells, plated using cells recovered from a minimum of 2 individual mice per condition. Colony-forming capacity for AML 4 was not detectable with up to 150,000 human cells assayed. ***$p \leq 0.0001$ (2-way factorial ANOVA). There was no significant interaction effect between patient sample and treatment group.
Data are summarized as means ± SEMs.
See also Figures S1 and S2 and Tables S1–S4.

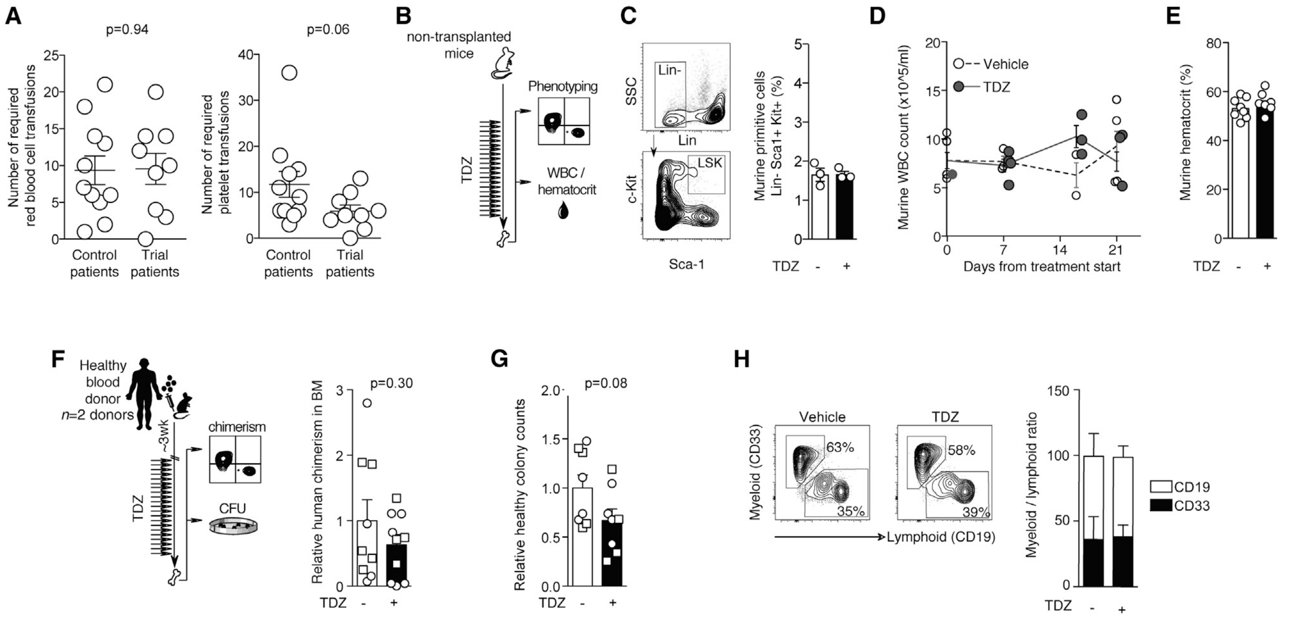

Figure 2. TDZ-induced suppression of progenitor activity is exclusive to AML

(A) Number of required red blood cell or platelet transfusions for trial AML patients receiving TDZ together with intermediate dose cytarabine compared to age- and disease-matched control AML patients receiving standard re-induction chemotherapy with HiDAC, FLAG-IDA, or idarubicin plus cytarabine ("control patients"). Data points compare 9 on-study patients treated with 21 days of TDZ and cytarabine (co-administered on days 6–10), versus 11 control AML patients treated with standard re-induction therapy. Transfusions were enumerated for a duration of 36 days for trial patients and 28 days for control patients. Trial patients 3T, 8T, 12T, and 13T were excluded from this analysis as they missed a significant portion of the 21-day TDZ treatment.

(B–E) Non-transplanted mice were treated with TDZ or 30% captisol (vehicle control, "−") for 21 days *in vivo*, followed by flow cytometric analysis of endogenous healthy stem and progenitor cells (C), white blood cell counts (WBCs) (D), and hematocrit levels (E).

(C) FACS plots showing the gating strategy for the murine stem and progenitor fraction Lin^- $Sca\text{-}1^+$ Kit^+ (LSK) within mouse BM. LSK frequencies were quantified after *in vivo* treatment with TDZ versus vehicle control ("−"). Symbols represent individual mice.

(D) Murine WBC counts throughout 21 days of exposure to TDZ or vehicle control *in vivo*. Symbols represent mean of 2 readings per individual mouse.

(E) Murine hematocrit levels following a 21-day administration of TDZ or vehicle control *in vivo*. Symbols represent average of 3 readings per individual mouse.

(F) Healthy human xenografts established from 2 distinct Lin^- CB samples were treated with TDZ (+) or vehicle control (−) *in vivo*, followed by analysis of human chimerism ($hCD45^+$) (F, right) and progenitor activity (G). Symbols represent individual recipient mice (circles, healthy donor 1; squares, healthy donor 2). p = 0.30, unpaired t test.

(G) Progenitor capacity in healthy donor xenografts isolated from recipient mouse BM; 10,000 human cells were interrogated in semisolid media; p = 0.08, unpaired t test.

(H) Ratio of myeloid:lymphoid cells within healthy xenografts after exposure to TDZ (+) or vehicle control (−) *in vivo*; n = 9–10 individual mice engrafted with human cells from n = 2 healthy donors. Representative FACS plots demonstrate myeloid (CD33) and lymphoid (CD19) populations.

Data are summarized as means ± SEMs.

limiting the duration and frequency of treatment that can be applied. A key goal of targeted cancer therapy is to achieve greater therapeutic specificity toward diseased cells, with fewer consequences to healthy tissue function.[1] Ideally, this would enable outpatient forms of treatment that can be tolerated as long-term oral medications.[26]

In the clinical trial setting, we previously reported that platelet and neutrophil counts remain stable over 5 consecutive days of DRD2 antagonism with TDZ,[19] indicating a lack of acute toxicity to normal hematopoiesis. Beyond this 5-day period of TDZ monotherapy, the same trial patients continued to receive oral TDZ daily, concurrent with standard cytarabine chemotherapy which was introduced on day 6 of the regimen. Over the full treatment course, consisting of 21-day TDZ administration q6h, the requirement for transfusion support was comparable to that of patients who received standard chemotherapy alone (Figure 2A). In fact, the number of required platelet transfusions was slightly reduced in TDZ trial patients, and this difference approached statistical significance (Figure 2A). This provides initial evidence that prolonged TDZ treatment can be safely and practically coupled with conventional chemotherapy regimens and does not cause additional harm to the hematopoietic system.

To more directly evaluate the long-term safety of TDZ as a single agent, we performed controlled *in vivo* experimentation in healthy non-leukemic mice (Figure 2B). Relative to vehicle control treatment, 21-day TDZ administration had no negative effects on murine hematopoietic stem/progenitor populations (Figure 2C), leukocyte counts (Figure 2D), or red blood cells (Figure 2E). Furthermore, the TDZ treatment regimen had no adverse effects against healthy human hematopoiesis in cord blood-derived xenografts, as measured by overall human chimerism (Figure 2F), healthy progenitor capacity (Figure 2G), or mature lineage composition (Figure 2H). These data

indicate the specificity of TDZ for leukemic, not normal hematopoiesis.

DRD cell surface phenotype predicts AML progenitor response to DRD-modulating agents

While limited by sample size, we previously established that clinical responsiveness to TDZ was associated with higher baseline DRD2 expression levels in leukemic blasts.[19] This observation motivated a systematic interrogation of responsiveness to TDZ based on patient-specific DRD2 expression, to evaluate a precision medicine strategy for future DRD-directed therapies. DRD2 expression levels were measured in an extended set of patient samples, including newly diagnosed cases of AML (Table S1). Since leukemic progenitors represented a critical disease subset affected by TDZ treatment (Figures 1B and 1H), we prioritized our phenotypic characterization of DRD2 to the CD34$^+$ subfraction of cells that enriches for progenitor activity (Figure S3A). Healthy CD34$^+$ cells were used to establish a threshold for aberrant DRD2 levels in AML patient samples. This threshold-based criterion allowed the segregation of AML patients into 2 categories; DRD2$^+$ patients versus DRD2^{lo} patients with levels similar to healthy controls (Figure 3A). Within our patient cohort, excessive DRD2 expression was related to greater disease severity as predicted by European LeukemiaNet (ELN) stratification.[27] Patients in the low-risk prognostic group expressed healthy ranges of DRD2 expression, whereas elevated DRD2 levels were only seen among patients with intermediate- or high-risk disease (Figure 3B). This provides a preliminary basis to suggest that DRD2$^+$ phenotypes are clinically significant and may be associated with underlying disease genotypes.

Functional anti-leukemic effects were tested using 8 DRD2$^+$ AML patient samples versus 5 DRD2$^{lo/-}$ controls (comprising 2 DRD2$^{lo/-}$ AML patients and 3 healthy donor samples; Figure 3C). Semisolid colony-forming unit (CFU) assays revealed that *in vitro* TDZ exposure suppressed both the number (Figure 3C; Table S5) and proliferative capacity of leukemic progenitors (Figures 3D and S3B) exclusively in DRD2$^+$ patients. In contrast, progenitor frequencies of DRD2$^{lo/-}$ AML patients remained minimally affected by TDZ, similar to the lack of response seen in healthy progenitor cells (Figures 3C and 3D). These findings suggest that pre-screening patients for DRD2 expression would be a valuable strategy to prospectively identify the individuals most likely to benefit from treatment with TDZ or similar targeted molecules.

To more directly address the relationship between DRD2 expression and self-renewal, we used fluorescence-activated cell sorting (FACS) to purify human AML cells into DRD2$^+$ versus DRD2$^-$ fractions, followed by functional evaluation by xenotransplantation. DRD2$^+$ cells efficiently produced human leukemic grafts in immunodeficient mice (Figures 3E and S3B), demonstrating clear self-renewal activity among DRD2$^+$ AML cells. DRD2$^-$ cells also possessed leukemia initiation capacity (Figures 3E and S3C); however, the absence of DRD2 signal by flow cytometry does not rule out the presence of intracellular DRD2 expression. It has been well established that DRD2 protein is not always localized at the cell surface and can transit to intracellular endosomal compartments, a common feature of G protein-coupled receptors.[28–31] Consistent with this phenomenon, we detected considerable DRD2 protein expression in bulk cellular lysates from cells that were purified based on DRD2 negativity at the cell surface (Figure 3F). Active signal transduction was also evident in the expression of the downstream signaling mediator, phosphorylated cyclic AMP (cAMP) response element binding protein (pCREB; Figure 3F). This suggests that DRD2$^-$ sorted cells do not represent a stable DRD2$^-$ population, precluding meaningful quantitative comparisons relative to DRD2$^+$ sorted subsets. This is similar to other G-coupled proteins used for hematopoietic cell surface identification (e.g., CXCR4) that failed to resolve clear mechanisms of self-renewal activity in hematopoietic stem cells (HSCs).[32–34] Importantly, patients that were considered DRD2$^{lo/-}$ by cell surface protein remained DRD2^{lo} when assayed for total DRD2 protein content by western blot, as the 2 measurements were highly correlated ($p = 0.009$; Figure S3D). This suggests that internalized reservoirs of DRD2 protein are more likely to exist in patients with elevated expression of DRD2 at the cell surface level.

Next, we more thoroughly explored the functional biology of DRD2$^+$ AML using alternate inhibitors of DRD2 signaling, to test the robustness of this candidate therapeutic target. Similar to TDZ, DRD2 antagonists fluphenazine dihydrochloride[35] and domperidone[36] independently suppressed leukemic progenitor activity, providing convergent evidence that DRD2 is a biologically meaningful target in AML (Figure S3E). Conversely, DRD2 stimulation, by its natural ligand, dopamine, augmented leukemic progenitor capacity (Figures 3G and 3H). This finding suggests that dopamine availability is likely to affect disease behavior in patients, a possibility that deserves attention given our related observation that the plasma of AML patients ($n = 11$) manifests elevated dopamine levels compared to healthy control plasma samples ($n = 19$) (Figure 3I). Overall, our combined results suggest that DRD2 dependence is of functional consequence to leukemic progenitor activity and that dopaminergic signaling presents a tunable axis for malignant transformation in a subset of DRD2$^+$ AML patients.

DRD signaling provides a selective axis to initiate maturation programs in AML progenitors

Downstream DRD signaling has been traditionally characterized to operate through adenylyl cyclase (AC)-mediated control of cAMP levels, where DRDs play opposing roles to regulate this network.[10,37] Within CD34$^+$ subsets of human AML, DRD1 and DRD2 are commonly co-expressed (Table S1). Although DRD1 expression levels often slightly exceed those of DRD2, DRD2 has a much higher binding affinity for dopamine ligand.[38] Upon ligand binding, DRD1 stimulation leads to AC activation and subsequent cAMP stimulation while DRD2 negatively regulates AC, thereby blocking cAMP induction.[10,37] Given that cAMP represents a central point of DRD pathway convergence, we explored its use as a molecular marker of targeted therapy response. Following *in vitro* culture with or without TDZ, we measured cAMP levels in the primary AML cells of patients from our Phase I clinical trial. In line with cell-intrinsic response patterns to TDZ, cAMP elevation was strictly observed in clinically responsive patients' samples (Figure 4A). Next, we used alternative tool compounds to further probe the mechanisms and effects of

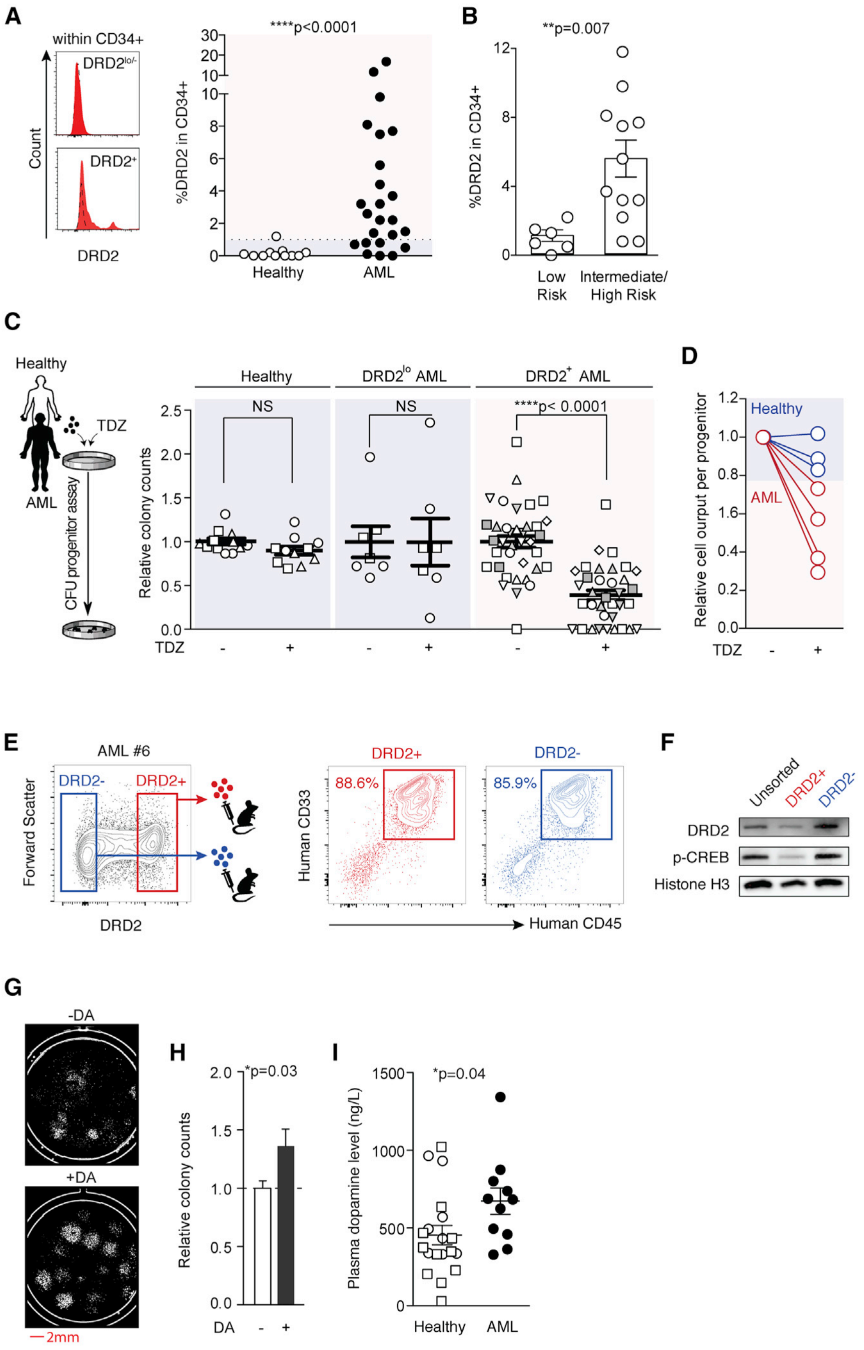

Figure 3. DRD2 expression profiles reliably predict functional response to DRD antagonism

(A) DRD2 expression patterns within leukemic CD34$^+$ cells. Dotted line represents FMO control (left). Comparison of DRD2 protein levels in CD34$^+$ cells of AML patient versus healthy donor samples (right). Healthy donor samples consist of cord blood (n = 3), adult mobilized peripheral blood (n = 3), and adult non-mobilized peripheral blood (n = 5). Blue versus red shading indicates the threshold of normal versus aberrant DRD2 levels. ****p $\leq$ 0.0001 (Mann-Whitney *U* test).

(B) DRD2 protein expression within CD34$^+$ subset of low versus intermediate-/high-risk AML patients based on ELN criteria.[27] Dots represent individual AML patients. **p = 0.006 (Mann-Whitney *U* test).

(C) Mononuclear cells (MNCs) isolated from healthy donors and AML patients were treated with TDZ or DMSO (vehicle control, "−") for 24 h and evaluated in progenitor CFU assays. Distinct shapes or colors indicate individual samples. n = 3–10 CFU wells per condition, ****p $\leq$ 0.0001 (unpaired t test). Source data can be found in Table S5.

(D) Proliferative capacity of leukemic versus healthy progenitor units was compared after *in vitro* exposure to TDZ for 24 h. Cell number output per colony was evaluated by custom scripts as a measure of proliferation.

(E) Representative FACS plots demonstrate gating strategy to purify DRD2$^+$ vs DRD2$^-$ human AML cells (left) and human leukemic chimerism in mice transplanted with 1 million DRD2$^+$ or DRD2$^-$ human AML cells.

(F) Western blot of DRD2, activated CREB (p-CREB at Ser-133), and histone H3 (loading control) in DRD2$^+$ versus DRD2$^-$ sorted fractions illustrated in (E).

(G) Representative whole-well CFU images after treatment with dopamine (DA) at physiological levels (10 nM) versus DMSO control (-DA).

(H) Progenitor cell activity was quantified in n = 6 distinct AML patients after treatment with physiological levels of DA (10–100 nM) relative to DMSO control. n = 2–3 CFU wells per AML sample. *p = 0.03 (unpaired t test).

(I) Circulating DA levels in healthy individuals (n = 8 healthy adult peripheral blood (PB) and 11 cord blood (CB) samples, as hollow circles and squares, respectively) versus n = 11 AML patients (black circles). *p = 0.04 (unpaired t test).

Data are summarized as means ± SEMs relative to vehicle control.

See also Figure S3 and Table S5.

DRD-mediated cAMP signaling on leukemic progenitor function. As would be expected based on classical DRD networks, an agonist to DRD1 (SKF 38393)[39] increased cAMP levels and also repressed leukemic progenitors (Figure 4B), similar to the effects of DRD2 antagonism. We additionally identified a specific DRD1 antibody that had agonist activity as seen by potent cAMP induction (Figure 4C) and subsequent activation of CREB (Figure 4D). Not only did αDRD1 treatment recapitulate the molecular hallmarks of TDZ treatment but it also suppressed leukemic progenitors in a similar fashion (Figure 4E). In contrast, dopamine ligand, which conversely had an activating effect on leukemic progenitors (Figures 3E and 3F), failed to induce cAMP (Figure S4A).

Beyond serving as a net indicator of DRD pathway activity, the involvement of cAMP also provides mechanistic insight to understand the anti-leukemic effects of TDZ, due to its established role in myeloid cell maturation.[40–42] Concomitant with the activation of cAMP, *in vitro* TDZ exposure led to AML cell maturation made apparent by morphology (Figures 4F and S4B),

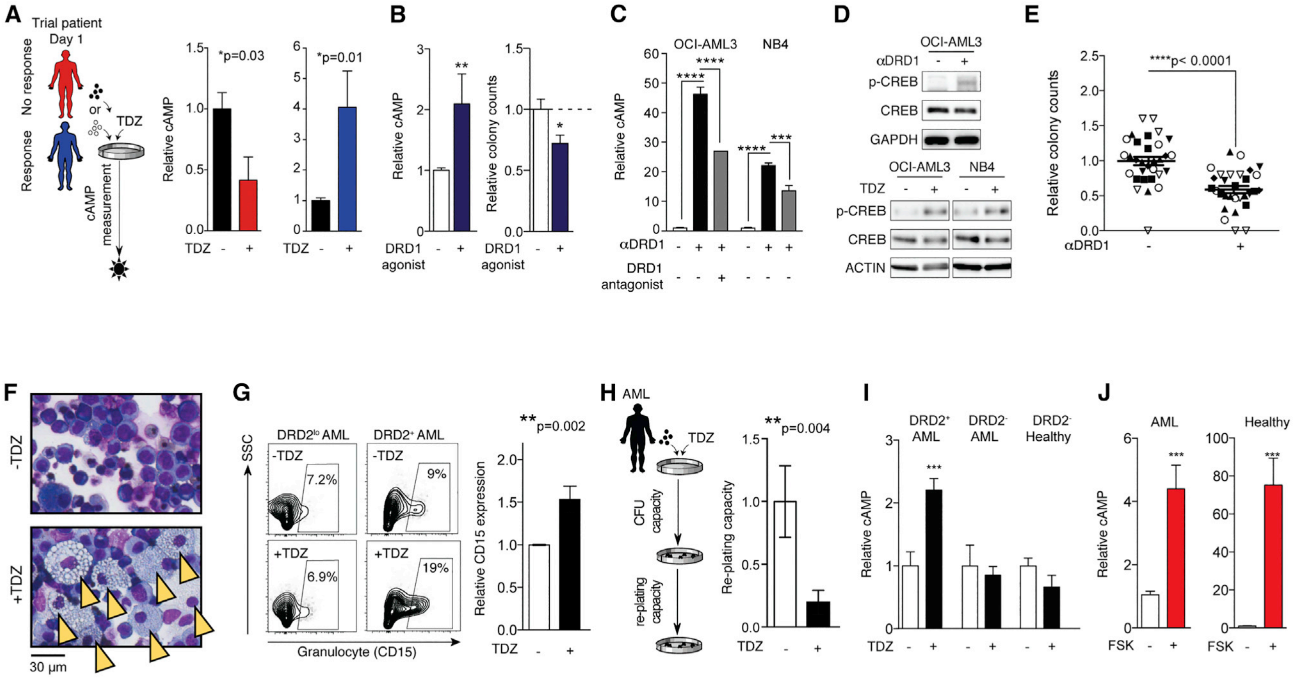

Figure 4. cAMP elevation is associated with leukemic progenitor suppression

(A) Trial patients (NCT02096289) were exposed to TDZ *in vitro*, followed by analysis of cAMP level changes. Trial patients with abundant cell numbers available were prioritized for this analysis, including patients 1T and 3T from non-responders, and patients 7T, 10T, and 11T for responders. n = 3–6 technical replicates per condition. $^{*}p \leq 0.05$ (unpaired t test).

(B) cAMP levels in response to DRD1 agonist (SKF 38393) relative to DMSO control. $n \geq 4$ replicates across OCI-AML3 and NB4 cell lines. $^{**}p = 0.008$ (Mann-Whitney *U* test). Progenitor response was evaluated after treatment with DRD1 agonist (SKF 38393) relative to DMSO control. n = 2–3 CFU replicates per AML sample (n = 5 AML samples total).

(C) cAMP levels in response to anti-DRD1 antibody alone or in combination with DRD1 antagonist (SCH 23390) in AML cell lines OCI-AML3 and NB4. n = 2–4 replicates per condition.

(D) Western blot of activated CREB (p-CREB at Ser-133) after exposure to anti-DRD1 antibody in OCI-AML3 cell line (top). Western blot of activated CREB (p-CREB at Ser-133) exposure to TDZ in OCI-AML3 and NB4 cell lines (bottom).

(E) MNCs isolated from healthy donors and AML patients were treated with anti-DRD1 antibody or immunoglobulin G (IgG) control ("–") for 30 min, and evaluated in progenitor CFU assays. Distinct shapes or colors indicate individual samples. n =3–7 CFU wells per condition, $^{****}p \leq 0.0001$ (unpaired t test).

(F) Cytospin preparations of AML cells from patient 2 after exposure to TDZ or vehicle control (DMSO). Yellow arrowheads indicate evidence of hematopoietic maturation (increased cell size, reduced nuclear:cytoplasmic ratio, increased cytoplasmic vacuolization).

(G) FACS plot showing expression of granulocytic cell marker (CD15) after *in vitro* exposure to TDZ or DMSO control ("-TDZ") in representative $DRD2^{lo}$ and $DRD2^{+}$ AML samples. CD15 frequencies were quantified for AMLs 1, 6, and 7 (n = 2 technical replicates per AML sample in each condition). $^{**}p = 0.002$ (Mann-Whitney *U* test).

(H) AML patient cells were treated with TDZ or DMSO for 24 h and evaluated in progenitor CFU assays, followed by analysis of re-plating capacity. $^{**}p = 0.004$ (unpaired t test).

(I) cAMP levels in response to TDZ relative to DMSO control. $DRD2^{+}$ AML includes AML 1, 6, OCI-AML3, and NB4. $DRD2^{-}$ AML and healthy controls include AML 12 and 3 CB samples, respectively. $n \geq 3$ replicates per condition. $^{***}p = 0.007$ (unpaired t test).

(J) cAMP levels in response to forskolin (FSK) relative to DMSO control. n = 6 replicates per condition, across 1 AML cell line and n = 2 healthy donor cells. $^{***}p \leq 0.0001$ (unpaired t test).

Data are summarized as means ± SEMs.

See also Figure S4.

immunophenotype[43] (Figure 4G), and a loss of self-renewal measured by progenitor re-plating capacity (Figure 4H). This induction of cellular maturation was accompanied by a mild reduction in DRD2 cell surface protein expression (Figure S4C), which we have also seen after *in vivo* TDZ treatment of AML xenografts.[25] These combined data collectively indicate that DRDs can be targeted to increase in cAMP levels in AML progenitors to induce maturation at the expense of progenitor capacity.

We next further explored the configuration of these networks in normal hematopoietic cells. Although healthy hematopoietic progenitors are competent to transduce cAMP signals, they lack the upstream DRD2 machinery required to mount a cAMP response to TDZ (Figure 3A). In line with being functionally inert to TDZ (Figures 3C and 3D), progenitor-enriched cells from healthy donors failed to show an elevation of cAMP upon *in vitro* TDZ treatment (Figure 4I). However, cAMP levels could be efficiently induced in healthy hematopoietic cells using the small molecule forskolin (FSK) that directly interacts with AC[39] and bypasses the need for DRD receptor engagement (Figure 4J). We suggest that while the cAMP circuitry is functionally

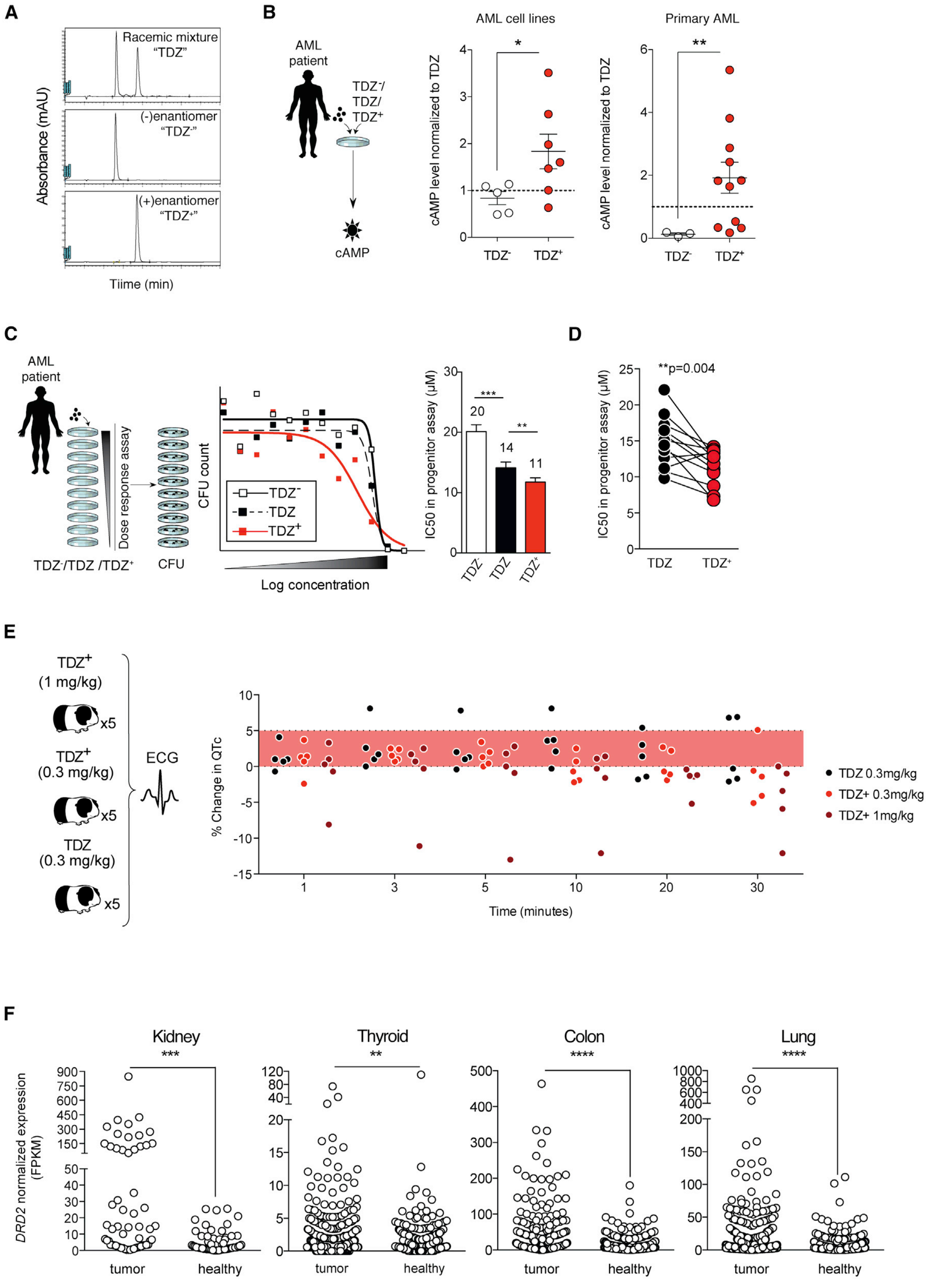

(legend on next page)

CellPress
OPEN ACCESS

intact in both healthy and leukemic hematopoiesis, the preferential presence of DRDs in AML provides an exclusive pharmacological gateway to the cAMP pathway in leukemic progenitors. We propose that this mechanism of action available through DRDs can be exploited selectively to target malignant hematopoiesis while sparing healthy hematopoiesis.

An enantiomer of TDZ displays a superior efficacy:risk ratio relative to TDZ

TDZ has been historically associated with cardiac and neurological toxicities,[20,21] and both types of adverse event were observed in AML patients at higher doses of TDZ.[19] As a result, a dose of 50 mg/kg q6h was determined to be the maximum tolerated dose for AML, limiting safe dose escalation to achieve optimal anti-leukemic potential. TDZ is a chiral molecule, meaning that it exists in 2 forms with mirror image stereochemistry (i.e., [+] and [−] enantiomers). Traditionally, TDZ has been clinically administered as an equimolar racemic mixture of (+) and (−) enantiomers, each with distinct risk profiles and pharmacological properties.[44] Considering that the positive enantiomer of TDZ is associated with a greater affinity for DRD2,[44] while the negative enantiomer is more strongly implicated in neurotoxic effects,[45] we sought an opportunity to refine the commercial TDZ compound and isolate the positive enantiomers (TDZ^+) toward a maximized potency:risk ratio. Using chiral separation, we segregated TDZ into high-purity positive and negative enantiomers (Figure 5A). Consistent with the relative DRD2 affinities reported for racemic TDZ and its 2 enantiomers,[44] TDZ^+, with a nearly 3-fold binding preference for DRD2, displayed a superior induction of cAMP levels relative to TDZ^- (Figure 5B). Intuitively, racemic TDZ showed intermediate potency relative to the 2 enantiomers (Figure 5B). Comparative analysis of racemic TDZ and both enantiomers in functional dose-response assays revealed that TDZ^+ achieved superior suppression of leukemic progenitor activity (Figures 5C and 5D). These effects remained restricted to a subset of AML patients expressing DRD2 (Figure S5A).

TDZ^+ has been associated with a reduced neurotoxic profile.[45] However, a concrete evaluation of this molecule in clinically relevant models of cardiac toxicity remains to be reported. Using a standardized guinea pig model for cardiovascular function, we optimized the dose of TDZ *in vivo* to achieve clinically relevant plasma levels of TDZ that lead to prolonged QT interval (QTc) events in humans. In contrast to its parent compound, TDZ^+ did not lead to prolonged QTc events in any of the guinea pigs assayed (n = 10), when administered at the same dose (Figure 5E). In fact, QTc intervals remained within safe ranges when TDZ^+ dosage was escalated by a factor of 3-fold (Figure 5E). Although QTc intervals were decreased in some guinea pigs following treatment with 1 mg/kg TDZ^+, this effect was not statistically significant (p = 0.21), and these changes remained within the ranges of other drugs that have been safely administered to humans.[46]

Given the demonstrated clinical relevance of leukemic progenitor assays (Figure 1) and the accepted predictive value of our cardiotoxicity model,[46] we have established a strong body of evidence that TDZ^+ offers superior clinical potential to racemic TDZ. By providing the dual benefit of increasing therapeutic potency while reducing toxic side effect profiles, TDZ^+ represents a promising lead compound for the future development of DRD-targeted therapies in AML and potentially a variety of cancers that also aberrantly express DRD2. By accessing public gene expression datasets including The Cancer Genome Atlas (TCGA) and Genotype-Tissue Expression (GTEx), we have detected the preferential expression of DRD2 among malignant tissues of kidney, thyroid, colon, and lung relative to matched healthy tissue samples (Figure 5F). This mirrors similar findings previously reported for tumors of breast,[16,48] brain,[13] and liver tissue origin. More important, elevated DRD2 expression was associated with a survival disadvantage for patients with kidney, endometrial, urothelial, and thyroid tumors (Figure S5B). This suggests that in addition to the clinical application of AML, the development of targeted DRD therapies may benefit other patient populations.

DISCUSSION

Early clinical trials in AML are challenged by the frequent requirement to restrict enrollment to relapsed or refractory cases of disease that have poor probabilities of response.[49] Therefore, it is important to carefully review preliminary signs of efficacy and identify opportunities for improvement when possible. The successful evolution of second-generation FLT3 inhibitors sets a strong standard for this approach[5] and should be used as a

Figure 5. TDZ^+ displays superior potency and reduced toxicity relative to TDZ

(A) Chiral separation of TDZ using supercritical fluid chromatography. Chromatograms show the first and second peaks, indicating the (−) enantiomer “TDZ^-” and (+) enantiomer “TDZ^+,” respectively. Purified enantiomers were evaluated for effects on cAMP levels (B), and in progenitor CFU assays (C and D).

(B) cAMP levels were evaluated after *in vitro* treatment with TDZ and its two enantiomers in AML cell lines (NB4 and OCI-AML3) and primary patient cells (AMLs 2, 9, and 27). Symbols represent individual CFU wells. *$p \leq 0.05$ and **$p \leq 0.01$ (unpaired t test).

(C) AML patient cells were exposed to TDZ and its 2 enantiomers for 24 h in a dose-response assay *in vitro*, and subsequently evaluated in progenitor CFU assays. Bar graphs summarize half-maximal inhibitory concentration (IC_{50}) in progenitor CFU assays performed with AML patient cells. **$p \leq 0.01$ and ***$p \leq 0.001$ (paired t test).

(D) Comparison of TDZ and TDZ^+ IC_{50} for individual AML patients in CFU assays (represented in C). **p = 0.004 (paired t test).

(E) A 30-min monitoring of QTc level changes after intravenous injection of TDZ and TDZ^+ in a guinea pig assay (n = 5 animals per cohort). QTc increases over 5% were considered indicators of safety risks.[46] No group averages were statistically different from baseline values (repeated-measures ANOVAs).

(F) *DRD2* transcript (Gene: 1813) was analyzed from TGCA (tumor and normal tissue) and GTEx (normal tissue) RNA-sequencing projects.[47] Data points represent normalized gene expression levels (fragments per kilobase of transcript per million mapped reads [FPKM]) for *DRD2* from individual cancer patients or healthy donors.[47] ***$p \leq 0.001$ and ****$p \leq 0.0001$ (Mann-Whitney *U* test), **p = 0.01 (Kolmogorov-Smirnov test).

Data are summarized as means ± SEMs.

See also Figure S5.

model for novel targeted therapies. Two fundamental goals of targeted therapy development are to identify specific patient subsets most likely to derive benefit and to maximize selectivity profiles for fewer adverse effects.[3] We have addressed both of these objectives in our current evaluation of DRD2 targeting to treat AML disease. Based on the application of cellular and molecular assays shown to correlate with clinical response, we build on findings from the first trial of a DRD-directed therapy in AML (NCT02096289) to present an alternative drug formulation that is more suitable for further clinical investigation. In conjunction, we have established objective evidence to recommend that DRD2^{+} patients should be prioritized for subsequent trials in the future. Across experimental systems as well as in a clinical setting, TDZ exposure reproducibly led to the rapid depletion of leukemic progenitor function exclusively in AML patients who expressed abnormal levels of DRD2.[19]

AML represents a prototypical disease for hierarchically organized cancers in which a tumor is initiated and propagated by primitive disease subfractions.[50] Nevertheless, realistic therapeutic strategies that disable these functionally primitive properties remain limited. We report that leukemic progenitor cell function can be efficiently blocked through the DRD pathway, acting through AC to increase cAMP levels, ultimately inducing a cellular maturation response. Given the short survival timelines of AML patients, it is highly desirable to develop new strategies to target this progenitor fraction, which possesses a capacity to promptly affect rates of downstream cell generation.[51,52] Our characterization of the preclinical activity of TDZ^{+} represents a valuable step toward this goal, as it demonstrates an optimized efficacy:risk ratio as a DRD2 pathway inhibitor. As an improved lead drug, TDZ^{+} offers more potent suppression of leukemic progenitor activity that is complemented by reduced neurotoxicity[45] and less cardiac liability, both of which presented safety challenges with the parent TDZ compound.[19]

Finally, our work highlights the need to further explore the source and physiological role of dopamine in leukemia disease biology. In addition to the nerve fiber innervation in the bone marrow (BM)[53] that provides a niche-based source of dopamine in the leukemic cell habitat, functional catecholamine synthesis machinery has been described within myeloid cells.[54] The latter finding suggests that leukemia cell-intrinsic dopamine production could reasonably contribute to elevated levels of dopamine that have been observed in AML patients by both ourselves and others.[55] We further propose that the role of dopamine should be investigated under conditions of regenerating disease post-chemotherapy, as residual leukemic cells have been found to further upregulate DRD2 expression after chemotherapy treatment.[25] This suggests that the DRD pathway may become vital to regenerating leukemic cells. Beyond AML, multiple human cancers have been reported to aberrantly express DRD upon transformation,[13,16,48,56] a phenomenon that has been repeatedly associated with poor disease prognosis in a number of cancer types.[13,16] Despite the recurrent presence of DRDs in transformed cells and their implication in disease outcomes, their biology in the context of tissue transformation and neoplastic cell behavior remains poorly characterized. Our study reveals a role for DRDs within transformed hematopoiesis in which they provide a selective access route to suppress leukemic progenitors. This establishes a framework for the generation of DRD antagonists for potential therapeutic use in a broad range of cancers that acquire aberrant DRD expression beyond AML.

Limitations of study

AML disease genetics are widely heterogenous across patients.[57] Therefore, it is a considerable challenge to capture a comprehensive cross-section of the patient population that represents all of the various disease subtypes. In the present study, we have observed that high-risk forms of the disease are more likely to manifest excessive DRD2 expression. However, it will ultimately require additional highly powered studies to determine whether particular cytogenetic or somatic mutations specifically correlate with elevated DRD2 levels.

The subcellular localization of DRD2 protein also complicates straightforward interpretation of its role in AML disease biology. While our findings suggest that TDZ responsiveness depends on cell surface expression of DRD2, it is important to acknowledge that this phenotype does not necessarily represent a stable population of cells. This is because DRD2 is known to dynamically cycle between the cell surface and intracellular endosomal compartments.[28–31] We predict that as cells transition in and out of surface-expressing states over time, sustained therapeutic targeting is likely to affect a larger population of cells than we would predict by examining DRD2 surface levels in a single static analysis. Receptor internalization/cycling also limits the ability to accurately evaluate LSC content in DRD2^{+} versus DRD2^{-} subsets by prospective cell purification, due to the fact that DRD2^{-} cells defined by surface expression may still contain DRD2 protein in intracellular locations. This is an implication that would also extend to other receptors in the GPCR superfamily such as well-known hematopoietic regulator CXCR4.[33,34,58,59] In addition to this complexity, we cannot rule out the possibility that there may be relevant interactions between DRD2^{+} and DRD2^{-} cells that could contribute to the clinical activity observed upon TDZ treatment. This will require further detailed study to better understand the role of DRD2 in AML that will likely inform the significance of DRD2 acquisition in other human cancers identified here.

STAR★METHODS

Detailed methods are provided in the online version of this paper and include the following:

- KEY RESOURCES TABLE
- RESOURCE AVAILABILITY
  - Lead contact
  - Materials availability
  - Data and code availability
- EXPERIMENTAL MODEL AND SUBJECT DETAILS
  - Cell lines
  - Primary human hematopoietic samples and AML cell lines
  - Murine recipients and xenograft assays
- METHOD DETAILS

- ○ Fluorescence-activated cell sorting and flow cytometric analysis
- ○ *In vitro* cell culture
- ○ Cyclic AMP measurement assay
- ○ Methylcellulose colony forming unit assay and CFU imaging
- ○ Western blot analysis
- ○ Measurement of dopamine levels in human plasma
- ○ Chiral separation of TDZ enantiomers
- ○ Analysis of AML-specific aberrations
- ○ Small mammal evaluation of QTc
- ○ RNA purification, PCR and Affymetrix
- QUANTIFICATION AND STATISTICAL ANALYSIS
  - ○ DRD gene expression in public datasets
  - ○ Statistics

SUPPLEMENTAL INFORMATION

Supplemental Information can be found online at https://doi.org/10.1016/j.xcrm.2021.100202.

ACKNOWLEDGMENTS

The authors would like to thank the patients and donors of clinical specimens. We would like to thank Dr. Mark Levine from the Department of Oncology, McMaster University, for initiating and providing oversight of clinical trial NCT02096289, in which samples were used for the validation of findings presented in this study. We would also like to acknowledge Dr. Eleftherios Sachlos, Dr. Zoya Shapovalova, Monica Graham, Dr. Kennedy Makondo, and Jamie D. McNicol for their insights, technical assistance, and contribution to the work. We thank Dr. Ram Mishra and Asim H. Siddiqui in the Department of Psychiatry and Behavioral Neuroscience, McMaster University, for the high-performance liquid chromatography (HPLC) analysis, and Dr. Fanny L. Casado for assistance in dopamine measurement in human plasma samples. We also thank the London Regional Genomics Center for running the Affymetrix arrays and The Centre for Applied Genomics (TCAG) at the Hospital for Sick Children for performing AML-associated mutations analysis. This study was supported by the Canadian Cancer Society Research Institute (CCS Impact grant # 706694) and the Braley Foundation (to M.B.) and Jan's Graduate Scholarships in Stem Cell Research (to L.A.). M.B. is supported by the Canadian Chair Program, and holds the Canada Research Chair in Human Stem Cell Biology and Michael DeGroote Chair in Human Stem Cells.

AUTHOR CONTRIBUTIONS

L.A., A.L.B., Y.D.B., J.D.L., J.L.G.R., M.N., D.P.P., J.C.R., and R.R.M. performed the experiment design and data collection, analysis, and interpretation. B.L., A.X., and R.F. provided the study material and insights. M.B. led the study conception, design, manuscript writing, and provided final approval.

DECLARATION OF INTERESTS

The authors declare no competing interests.

Received: April 8, 2020
Revised: November 2, 2020
Accepted: January 19, 2021
Published: February 16, 2021

REFERENCES

1. Crisci, S., Amitrano, F., Saggese, M., Muto, T., Sarno, S., Mele, S., Vitale, P., Ronga, G., Berretta, M., and Di Francia, R. (2019). Overview of Current Targeted Anti-Cancer Drugs for Therapy in Onco-Hematology. Medicina (Kaunas) *55*, 414.
2. Estey, E.H. (2018). Acute myeloid leukemia: 2019 update on risk-stratification and management. Am. J. Hematol. *93*, 1267–1291.
3. Gao, W., and Estey, E. (2015). Moving toward targeted therapies in acute myeloid leukemia. Clin. Adv. Hematol. Oncol. *13*, 748–754.
4. Fleck, R., and Bach, D. (2012). Trends in Personalized Therapies in Oncology: The (Venture) Capitalist's Perspective. J. Pers. Med. *2*, 15–34.
5. Wander, S.A., Levis, M.J., and Fathi, A.T. (2014). The evolving role of FLT3 inhibitors in acute myeloid leukemia: quizartinib and beyond. Ther. Adv. Hematol. *5*, 65–77.
6. Zahreddine, H.A., Culjkovic-Kraljacic, B., Assouline, S., Gendron, P., Romeo, A.A., Morris, S.J., Cormack, G., Jaquith, J.B., Cerchietti, L., Cocolakis, E., et al. (2014). The sonic hedgehog factor GLI1 imparts drug resistance through inducible glucuronidation. Nature *511*, 90–93.
7. Sawaguchi, T., and Goldman-Rakic, P.S. (1991). D1 dopamine receptors in prefrontal cortex: involvement in working memory. Science *251*, 947–950.
8. Luchsinger, A., Velasco, M., Urbina, A., Morillo, J., Romero, E., Alvarez, R., and Pieretti, O.H. (1992). Comparative effects of dopaminergic agonists on cardiovascular, renal, and renin-angiotensin systems in hypertensive patients. J. Clin. Pharmacol. *32*, 55–60.
9. Kebabian, J.W., and Greengard, P. (1971). Dopamine-sensitive adenyl cyclase: possible role in synaptic transmission. Science *174*, 1346–1349.
10. Kebabian, J.W., and Calne, D.B. (1979). Multiple receptors for dopamine. Nature *277*, 93–96.
11. Seeman, P., Lee, T., Chau-Wong, M., and Wong, K. (1976). Antipsychotic drug doses and neuroleptic/dopamine receptors. Nature *261*, 717–719.
12. Seeman, P., and Lee, T. (1975). Antipsychotic drugs: direct correlation between clinical potency and presynaptic action on dopamine neurons. Science *188*, 1217–1219.
13. Dolma, S., Selvadurai, H.J., Lan, X., Lee, L., Kushida, M., Voisin, V., Whetstone, H., So, M., Aviv, T., Park, N., et al. (2016). Inhibition of Dopamine Receptor D4 Impedes Autophagic Flux, Proliferation, and Survival of Glioblastoma Stem Cells. Cancer Cell *29*, 859–873.
14. Huang, X., He, Y., Dubuc, A.M., Hashizume, R., Zhang, W., Reimand, J., Yang, H., Wang, T.A., Stehbens, S.J., Younger, S., et al. (2015). EAG2 potassium channel with evolutionarily conserved function as a brain tumor target. Nat. Neurosci. *18*, 1236–1246.
15. Cheng, H.W., Liang, Y.H., Kuo, Y.L., Chuu, C.P., Lin, C.Y., Lee, M.H., Wu, A.T., Yeh, C.T., Chen, E.I., Whang-Peng, J., et al. (2015). Identification of thioridazine, an antipsychotic drug, as an antiglioblastoma and anticancer stem cell agent using public gene expression data. Cell Death Dis. *6*, e1753.
16. Sachlos, E., Risueño, R.M., Laronde, S., Shapovalova, Z., Lee, J.H., Russell, J., Malig, M., McNicol, J.D., Fiebig-Comyn, A., Graham, M., et al. (2012). Identification of drugs including a dopamine receptor antagonist that selectively target cancer stem cells. Cell *149*, 1284–1297.
17. Nagel, D., Spranger, S., Vincendeau, M., Grau, M., Raffegerst, S., Kloo, B., Hlahla, D., Neuenschwander, M., Peter von Kries, J., Hadian, K., et al. (2012). Pharmacologic inhibition of MALT1 protease by phenothiazines as a therapeutic approach for the treatment of aggressive ABC-DLBCL. Cancer Cell *22*, 825–837.
18. Malta, T.M., Sokolov, A., Gentles, A.J., Burzykowski, T., Poisson, L., Weinstein, J.N., Kamińska, B., Huelsken, J., Omberg, L., Gevaert, O., et al.; Cancer Genome Atlas Research Network (2018). Machine Learning Identifies Stemness Features Associated with Oncogenic Dedifferentiation. Cell *173*, 338–354.e15.
19. Aslostovar, L., Boyd, A.L., Almakadi, M., Collins, T.J., Leong, D.P., Tirona, R.G., Kim, R.B., Julian, J.A., Xenocostas, A., Leber, B., et al. (2018). A phase 1 trial evaluating thioridazine in combination with cytarabine in patients with acute myeloid leukemia. Blood Adv. *2*, 1935–1945.

20. Reilly, J.G., Ayis, S.A., Ferrier, I.N., Jones, S.J., and Thomas, S.H. (2000). QTc-interval abnormalities and psychotropic drug therapy in psychiatric patients. Lancet *355*, 1048–1052.

21. Menkes, D.B., and Knight, J.C. (2002). Cardiotoxicity and prescription of thioridazine in New Zealand. Aust. N. Z. J. Psychiatry *36*, 492–498.

22. Pollyea, D.A., Gutman, J.A., Gore, L., Smith, C.A., and Jordan, C.T. (2014). Targeting acute myeloid leukemia stem cells: a review and principles for the development of clinical trials. Haematologica *99*, 1277–1284.

23. Hu, Y., and Smyth, G.K. (2009). ELDA: extreme limiting dilution analysis for comparing depleted and enriched populations in stem cell and other assays. J. Immunol. Methods *347*, 70–78.

24. Eppert, K., Takenaka, K., Lechman, E.R., Waldron, L., Nilsson, B., van Galen, P., Metzeler, K.H., Poeppl, A., Ling, V., Beyene, J., et al. (2011). Stem cell gene expression programs influence clinical outcome in human leukemia. Nat. Med. *17*, 1086–1093.

25. Boyd, A.L., Aslostovar, L., Reid, J., Ye, W., Tanasijevic, B., Porras, D.P., Shapovalova, Z., Almakadi, M., Foley, R., Leber, B., et al. (2018). Identification of Chemotherapy-Induced Leukemic-Regenerating Cells Reveals a Transient Vulnerability of Human AML Recurrence. Cancer Cell *34*, 483–498.e5.

26. Perl, A.E. (2017). The role of targeted therapy in the management of patients with AML. Blood Adv. *1*, 2281–2294.

27. Döhner, H., Estey, E.H., Amadori, S., Appelbaum, F.R., Büchner, T., Burnett, A.K., Dombret, H., Fenaux, P., Grimwade, D., Larson, R.A., et al.; European LeukemiaNet (2010). Diagnosis and management of acute myeloid leukemia in adults: recommendations from an international expert panel, on behalf of the European LeukemiaNet. Blood *115*, 453–474.

28. Calebiro, D., and Godbole, A. (2018). Internalization of G-protein-coupled receptors: implication in receptor function, physiology and diseases. Best Pract. Res. Clin. Endocrinol. Metab. *32*, 83–91.

29. Guo, N., Guo, W., Kralikova, M., Jiang, M., Schieren, I., Narendran, R., Slifstein, M., Abi-Dargham, A., Laruelle, M., Javitch, J.A., and Rayport, S. (2010). Impact of D2 receptor internalization on binding affinity of neuroimaging radiotracers. Neuropsychopharmacology *35*, 806–817.

30. Lee, S.A., Suh, Y., Lee, S., Jeong, J., Kim, S.J., Kim, S.J., and Park, S.K. (2017). Functional expression of dopamine D2 receptor is regulated by tetraspanin 7-mediated postendocytic trafficking. FASEB J. *31*, 2301–2313.

31. Skinbjerg, M., Liow, J.S., Seneca, N., Hong, J., Lu, S., Thorsell, A., Heilig, M., Pike, V.W., Halldin, C., Sibley, D.R., and Innis, R.B. (2010). D2 dopamine receptor internalization prolongs the decrease of radioligand binding after amphetamine: a PET study in a receptor internalization-deficient mouse model. Neuroimage *50*, 1402–1407.

32. Peled, A., Petit, I., Kollet, O., Magid, M., Ponomaryov, T., Byk, T., Nagler, A., Ben-Hur, H., Many, A., Shultz, L., et al. (1999). Dependence of human stem cell engraftment and repopulation of NOD/SCID mice on CXCR4. Science *283*, 845–848.

33. Rosu-Myles, M., Gallacher, L., Murdoch, B., Hess, D.A., Keeney, M., Kelvin, D., Dale, L., Ferguson, S.S., Wu, D., Fellows, F., and Bhatia, M. (2000). The human hematopoietic stem cell compartment is heterogeneous for CXCR4 expression. Proc. Natl. Acad. Sci. USA *97*, 14626–14631.

34. Sand, L.G.L., Buckle, T., van Leeuwen, F.W.B., Corver, W.E., Kruisselbrink, A.B., Jochemsen, A.G., Hogendoorn, P.C.W., and Szuhai, K. (2017). Fluorescent CXCR4 targeting peptide as alternative for antibody staining in Ewing sarcoma. BMC Cancer *17*, 383.

35. Seeman, P., and Ulpian, C. (1983). Neuroleptics have identical potencies in human brain limbic and putamen regions. Eur. J. Pharmacol. *94*, 145–148.

36. Fontaine, R., Affaticati, P., Yamamoto, K., Jolly, C., Bureau, C., Baloche, S., Gonnet, F., Vernier, P., Dufour, S., and Pasqualini, C. (2013). Dopamine inhibits reproduction in female zebrafish (Danio rerio) via three pituitary D2 receptor subtypes. Endocrinology *154*, 807–818.

37. Beaulieu, J.M., and Gainetdinov, R.R. (2011). The physiology, signaling, and pharmacology of dopamine receptors. Pharmacol. Rev. *63*, 182–217.

38. Reinart-Okugbeni, R., Ausmees, K., Kriis, K., Werner, F., Rinken, A., and Kanger, T. (2012). Chemoenzymatic synthesis and evaluation of 3-azabicyclo[3.2.0]heptane derivatives as dopaminergic ligands. Eur. J. Med. Chem. *55*, 255–261.

39. Kinoshita, S., Sidhu, A., and Felder, R.A. (1989). Defective dopamine-1 receptor adenylate cyclase coupling in the proximal convoluted tubule from the spontaneously hypertensive rat. J. Clin. Invest. *84*, 1849–1856.

40. Stegmaier, K., Ross, K.N., Colavito, S.A., O'Malley, S., Stockwell, B.R., and Golub, T.R. (2004). Gene expression-based high-throughput screening(GE-HTS) and application to leukemia differentiation. Nat. Genet. *36*, 257–263.

41. Tortora, G., Clair, T., Katsaros, D., Ally, S., Colamonici, O., Neckers, L.M., Tagliaferri, P., Jahnsen, T., Robins, R.K., and Cho-Chung, Y.S. (1989). Induction of megakaryocytic differentiation and modulation of protein kinase gene expression by site-selective cAMP analogs in K-562 human leukemic cells. Proc. Natl. Acad. Sci. USA *86*, 2849–2852.

42. Brodsky, A., Davio, C., Shayo, C., Lemos Legnazzi, B., Barbosa, M., Lardo, M., Morelli, A., Baldi, A., Sanchez Avalos, J.C., and Rivera, E. (1998). Forskolin induces U937 cell line differentiation as a result of a sustained cAMP elevation. Eur. J. Pharmacol. *350*, 121–127.

43. Pabst, C., Krosl, J., Fares, I., Boucher, G., Ruel, R., Marinier, A., Lemieux, S., Hébert, J., and Sauvageau, G. (2014). Identification of small molecules that support human leukemia stem cell activity ex vivo. Nat. Methods *11*, 436–442.

44. Svendsen, C.N., Froimowitz, M., Hrbek, C., Campbell, A., Kula, N., Baldessarini, R.J., Cohen, B.M., Babb, S., Teicher, M.H., and Bird, E.D. (1988). Receptor affinity, neurochemistry and behavioral characteristics of the enantiomers of thioridazine: evidence for different stereoselectivities at D1 and D2 receptors in rat brain. Neuropharmacology *27*, 1117–1124.

45. Jortani, S.A., Valentour, J.C., and Poklis, A. (1994). Thioridazine enantiomers in human tissues. Forensic Sci. Int. *64*, 165–170.

46. Yao, X., Anderson, D.L., Ross, S.A., Lang, D.G., Desai, B.Z., Cooper, D.C., Wheelan, P., McIntyre, M.S., Bergquist, M.L., MacKenzie, K.I., et al. (2008). Predicting QT prolongation in humans during early drug development using hERG inhibition and an anaesthetized guinea-pig model. Br. J. Pharmacol. *154*, 1446–1456.

47. Wang, Q., Armenia, J., Zhang, C., Penson, A.V., Reznik, E., Zhang, L., Minet, T., Ochoa, A., Gross, B.E., Iacobuzio-Donahue, C.A., et al. (2018). Unifying cancer and normal RNA sequencing data from different sources. Sci. Data *5*, 180061.

48. Borcherding, D.C., Tong, W., Hugo, E.R., Barnard, D.F., Fox, S., LaSance, K., Shaughnessy, E., and Ben-Jonathan, N. (2016). Expression and therapeutic targeting of dopamine receptor-1 (D1R) in breast cancer. Oncogene *35*, 3103–3113.

49. Estey, E., Levine, R.L., and Löwenberg, B. (2015). Current challenges in clinical development of "targeted therapies": the case of acute myeloid leukemia. Blood *125*, 2461–2466.

50. Lapidot, T., Sirard, C., Vormoor, J., Murdoch, B., Hoang, T., Caceres-Cortes, J., Minden, M., Paterson, B., Caligiuri, M.A., and Dick, J.E. (1994). A cell initiating human acute myeloid leukaemia after transplantation into SCID mice. Nature *367*, 645–648.

51. Sun, J., Ramos, A., Chapman, B., Johnnidis, J.B., Le, L., Ho, Y.J., Klein, A., Hofmann, O., and Camargo, F.D. (2014). Clonal dynamics of native haematopoiesis. Nature *514*, 322–327.

52. Kim, S., Kim, N., Presson, A.P., Metzger, M.E., Bonifacino, A.C., Sehl, M., Chow, S.A., Crooks, G.M., Dunbar, C.E., An, D.S., et al. (2014). Dynamics of HSPC repopulation in nonhuman primates revealed by a decade-long clonal-tracking study. Cell Stem Cell *14*, 473–485.

53. Yamazaki, K., and Allen, T.D. (1991). Ultrastructural and morphometric alterations in bone marrow stromal tissue after 7 Gy irradiation. Blood Cells *17*, 527–549.

54. Flierl, M.A., Rittirsch, D., Nadeau, B.A., Chen, A.J., Sarma, J.V., Zetoune, F.S., McGuire, S.R., List, R.P., Day, D.E., Hoesel, L.M., et al. (2007). Phagocyte-derived catecholamines enhance acute inflammatory injury. Nature *449*, 721–725.

55. Singh, J.N., Chansouria, J.P., Singh, V.P., and Udupa, K.N. (1989). Blood bioamines, cortisol and aminoacid levels in leukemic patients. Indian J. Cancer *26*, 222–226.

56. Lu, M., Li, J., Luo, Z., Zhang, S., Xue, S., Wang, K., Shi, Y., Zhang, C., Chen, H., and Li, Z. (2015). Roles of dopamine receptors and their antagonist thioridazine in hepatoma metastasis. OncoTargets Ther. *8*, 1543–1552.

57. Papaemmanuil, E., Gerstung, M., Bullinger, L., Gaidzik, V.I., Paschka, P., Roberts, N.D., Potter, N.E., Heuser, M., Thol, F., Bolli, N., et al. (2016). Genomic Classification and Prognosis in Acute Myeloid Leukemia. N. Engl. J. Med. *374*, 2209–2221.

58. Fiegl, M., Samudio, I., Clise-Dwyer, K., Burks, J.K., Mnjoyan, Z., and Andreeff, M. (2009). CXCR4 expression and biologic activity in acute myeloid leukemia are dependent on oxygen partial pressure. Blood *113*, 1504–1512.

59. Ramakrishnan, R., Peña-Martínez, P., Agarwal, P., Rodriguez-Zabala, M., Chapellier, M., Högberg, C., Eriksson, M., Yudovich, D., Shah, M., Ehinger, M., et al. (2020). CXCR4 Signaling Has a CXCL12-Independent Essential Role in Murine MLL-AF9-Driven Acute Myeloid Leukemia. Cell Rep. *31*, 107684.

60. Boyd, A.L., Campbell, C.J., Hopkins, C.I., Fiebig-Comyn, A., Russell, J., Ulemek, J., Foley, R., Leber, B., Xenocostas, A., Collins, T.J., and Bhatia, M. (2014). Niche displacement of human leukemic stem cells uniquely allows their competitive replacement with healthy HSPCs. J. Exp. Med. *211*, 1925–1935.

61. Salci, K.R., Lee, J.H., Laronde, S., Dingwall, S., Kushwah, R., Fiebig-Comyn, A., Leber, B., Foley, R., Dal Cin, A., and Bhatia, M. (2015). Cellular Reprogramming Allows Generation of Autologous Hematopoietic Progenitors From AML Patients That Are Devoid of Patient-Specific Genomic Aberrations. Stem Cells *33*, 1839–1849.

62. Somervaille, T.C., and Cleary, M.L. (2006). Identification and characterization of leukemia stem cells in murine MLL-AF9 acute myeloid leukemia. Cancer Cell *10*, 257–268.

63. Langenkamp, U., Siegler, U., Jörger, S., Diermayr, S., Gratwohl, A., Kalberer, C.P., and Wodnar-Filipowicz, A. (2009). Human acute myeloid leukemia CD34+CD38- stem cells are susceptible to allorecognition and lysis by single KIR-expressing natural killer cells. Haematologica *94*, 1590–1594.

64. Benoit, Y.D., Lussier, C., Ducharme, P.A., Sivret, S., Schnapp, L.M., Basora, N., and Beaulieu, J.F. (2009). Integrin alpha8beta1 regulates adhesion, migration and proliferation of human intestinal crypt cells via a predominant RhoA/ROCK-dependent mechanism. Biol. Cell *101*, 695–708.

65. Uhlen, M., Zhang, C., Lee, S., Sjöstedt, E., Fagerberg, L., Bidkhori, G., Benfeitas, R., Arif, M., Liu, Z., Edfors, F., et al. (2017). A pathology atlas of the human cancer transcriptome. Science *357*, eaan2507.

STAR★METHODS

KEY RESOURCES TABLE

REAGENT or RESOURCE	SOURCE	IDENTIFIER
Antibodies		
V450 mouse anti-human CD45	BD Horizon	Cat#642275; RRID:AB_1645755
BV605 mouse anti-human CD45	BH Horizon	Cat#564048; RRID:AB_2744403
APC mouse anti-human CD33	BD PharMingen	Cat#551378; RRID:AB_398502
BV421 mouse anti-human CD33	BD Biosciences	Cat#565949; RRID:AB_2739413
APC mouse anti-human CD34	BD PharMingen	Cat#555824: RRID:AB_398614
PE mouse anti-human CD34	BD PharMingen	Cat#555822; RRID:AB_396151
FITC mouse anti-human CD15	BD Biosciences	Cat#555401; RRID:AB_395801
PE mouse anti-human CD15	Beckman Coulter	Cat#IM1954U; RRID:AB_10638572
Rabbit anti-human DRD2	Millipore	Cat#324393; RRID:AB_211787
Mouse anti-human DRD2	Santa Cruz	Cat#sc-5303; RRID:AB_668816
Rabbit anti-human DRD1	Millipore	Cat#324390; RRID:AB_564546
Donkey anti-mouse Alexa 647	Thermo Fisher	Cat#A31571; RRID:AB_162542
Donkey anti-mouse Alexa 555	Thermo Fisher	Cat#A31570; RRID:AB_2536180
Donkey anti-rabbit Alexa 647	Thermo Fisher	Cat#A31573; RRID:AB_2536183
Rabbit anti-phospho-CREB (Ser133)	Millipore	Cat#06-519; RRID:AB_310153
Mouse anti-CREB	Cell Signaling Technology	Cat#9104; RRID:AB_490881
Rabbit anti-human DRD2	Millipore	Cat# AB5084P; RRID:AB_2094980
Mouse anti-Actin	Millipore	Cat#MAB1501; RRID:AB_2223041
Mouse anti-GAPDH	Abcam	Cat#ab8245; RRID:AB_2107448
Rabbit anti-human Histone H3	Cell Signaling Technology	Cat#9715; RRID:AB_331563
Biological samples		
Primary AML patient samples	Juravinksi hospital and Cancer Centre London Health Sciences Centre	N/A
AML patient-derived xenografts	Juravinksi hospital and Cancer Centre London Health Sciences Centre	N/A
Healthy human donor hematopoietic samples	Labour and delivery clinic at McMaster Children's hospital Juravinksi hospital and Cancer Centre	N/A
Healthy human blood donor-derived xenografts	Labour and delivery clinic at McMaster Children's hospital Juravinksi hospital and Cancer Centre	N/A
Chemicals, peptides, and recombinant proteins		
7AAD	Beckman Coulter	Cat#A07704
Trypan Blue	GIBCO	Cat#15250061
Stem Cell Factor (SCF)	R&D Systems	Cat#255-SC/CF
Fms-related tyrosine kinase 3 ligand	R&D Systems	Cat#308-Fk/CF
Recombinant human Thrombopietin protein	R&D Systems	Cat#288-TP-005/CF
RPMI 1640 Medium	GIBCO	Cat#11875119
Fetal Bovine Serum	HyClone	Cat# SH3039603
Stemspan	Stem Cell Technologies	Cat#09650
Captisol	Ligand Pharmaceuticals	Cat#RC-0C7-020
Thioridazine hydrochloride	Sigma	Cat#T9025; CAS#130-61-0
(±)-SKF-38393 hydrochloride	Sigma	Cat#D047; CAS#62717-42-4
R(+)-SCH-23390 hydrochloride	Sigma	Cat#D054; CAS#125941-87-9

(*Continued on next page*)

Continued

REAGENT or RESOURCE	SOURCE	IDENTIFIER
Domperidone	Sigma	Cat#D122; CAS#57808-66-9
Fluphenazine dihydrochloride	Sigma	Cat#4765; CAS#146-56-5
Forskolin	Abcam	Cat#ab120058; CAS#66575-29-9
Fc receptor binding inhibitor	eBiosciences	Cat#14-9161-71
Donkey serum	Jackson ImmunoResearch Laboratories	Cat#017-000-121
Platinum SuperFi DNA Polymerase	Thermo Fisher	Cat#12351010
Critical commercial assays		
Total RNA purification kit	Norgen Biotek	Cat#37500
QIAamp DNA Micro kit	QIAGEN	Cat#56304
Methocult	Stemcell technologies	Cat#h4434
ddPCR Supermix for Probes	BioRad Laboratories	Cat#1863010
cAMP direct immunoassay kit	Millipore/Calbiochem	Cat# 116811
Shandon Kwik-Diff Stains	Thermo Fisher	Cat#9990700
Deposited data		
Raw Microarray data	This paper	GSE82057
Experimental models: cell lines		
Human: NB-4	DSMZ	Cat#ACC 207
Human: OCI-AML3	DSMZ	Cat#ACC 582
Experimental models: organisms/strains		
NOD.CB17-*Prkdc*^{scid}/J	The Jackson laboratory	RRID:IMSR_JAX:001303
NOD.Cg-*Prkdc*^{scid}Il2rg^{tm1wjl}/SzJ	The Jackson laboratory	RRID:IMSR_ARC:NSG JAX:05557
Oligonucleotides		
FLT3 exons 13-15 F: 5′-attgtcgttttaaccctgctaat −3′	This paper	N/A
FLT3 exons 13-15 R: 5-ttttgctaattccataagctgtt −3′	This paper	N/A
AUTS2 on 7q11.22	TaqMan (Thermo Fisher)	Cat#4400291; Assay ID Hs04327806_cn
RPPH1 on 21q21.3	TaqMan (Thermo Fisher)	Cat#4400291; Assay ID Hs05538458_cn
Software and algorithms		
FACSDiva	BD	https://www.bdbiosciences.com/us/instruments/research/software/flow-cytometry-acquisition
FlowJo10	FlowJo, LLC	https://www.flowjo.com
Prism v5.0a	Graphpad	https://www.graphpad.com/
Harmony High-Content Imaging and Analysis Software	PerkinElmer	https://www.perkinelmer.com/operetta-cls/harmony-software/
Columbus Image Data Storage and Analysis System	PerkinElmer	https://www.perkinelmer.com/product/imagedata-storage-and-analysis-system-columbus
Genomics Suite 6.6 software	Partek Inc.	https://www.partek.com/pgs
GSEA vs2.1.0	Broad Institute	http://www.gsea-msigdb.org/gsea/login.jsp
QuantaSoft Analysis Pro v1.0.596	BioRad Laboratories	https://www.bio-rad.com/

RESOURCE AVAILABILITY

Lead contact

Further information and requests for resources and reagents should be directed to and will be fulfilled by the Lead Contact, Mickie Bhatia (mbhatia@mcmaster.ca).

Materials availability

This study did not generate new unique reagents.

Data and code availability

Microarray data generated during this study can be accessed at GEO: GSE82057. Source data for Figure 1H are available in Table S4. Source data for Figure 3C are available in Table S5.

EXPERIMENTAL MODEL AND SUBJECT DETAILS

Cell lines

Leukemia cell lines OCI-AML3 and NB4 were purchased from DSMZ. OCI-AML3 was derived from a male patient with DNMT3A and NPM1 mutations and the NB4 cell line was derived from a female patient with a PML-RARA gene fusion as well as KRAS and TP53 mutations. Both cell lines were maintained in RPMI 1640 (VWR) supplemented with 10% FBS (HyClone).

Primary human hematopoietic samples and AML cell lines

All patients and healthy donors provided written informed consent, in accordance with Research Ethics Board-approved protocols at McMaster University and the London Health Sciences Centre. This study is fully compliant with all relevant ethical regulations regarding human participants. Primary leukemia samples were obtained from peripheral blood apheresis or BM aspirates of AML patients. Healthy hematopoietic cells were isolated from BM and mobilized peripheral blood (MPB) of adult donors, or from umbilical cord blood. The Labour and Delivery Clinic at the McMaster Children's Hospital provided healthy cord blood samples. Adult sources of hematopoietic cells were provided by the Juravinski Hospital and Cancer Centre, and London Health Sciences Centre (University of Western Ontario). Detailed clinical descriptions of AML patient samples are outlined in Table S1.

Once acquired from the clinic, primary hematopoietic samples were processed to isolate mononuclear cells (MNCs) as previously described.[16] Lineage depletion (Lin-) of CB and MPB samples was performed by magnetic cell separation using EasySep immunomagnetic cell separation (StemCell Technologies, Inc.).

Murine recipients and xenograft assays

NOD/Prkdc^{scid} or NSG mice were used as xenograft recipients. Mice were bred in a barrier facility and all experimental protocols were approved by the Animal Care Council of McMaster University. This study is fully compliant with all relevant ethical regulations regarding animal research. Both male and female mice were used throughout the study, however individual experiments exclusively involved either male or female recipients, to control for the influence of sex as a variable. For transplantation assays, 6-10 week-old mice were sublethally irradiated (350 rads) approximately 24 hours prior to intravenous injection of primary human samples[60] (~5 million AML mononuclear cells or 150,000 lineage depleted cord blood cells per mouse). For *in vivo* TDZ treatment, a daily dose of TDZ at 22.5 mg/ kg/ day that gives rise to clinically relevant plasma TDZ levels[25] was administered intraperitoneally for 21 days. 30% captisol (Ligand Pharmaceuticals) was administered in vehicle control-treated recipients. Weight was measured weekly to ensure that an appropriate dose per weight ratio was sustained throughout the treatment period. Mice were allocated to treatment groups based on chimerism levels assessed by BM aspiration prior to the treatment start, to ensure equalized engraftment levels across groups. If no initial assessment of chimerism was performed, mice were randomly allocated to experimental groups.

METHOD DETAILS

Fluorescence-activated cell sorting and flow cytometric analysis

Immunophenotyping of cell surface markers was carried out using CD45 (642275, clone 2D1, BD PharMingen; 564048, clone H130, BD Horizon), CD34 (555822, clone 581, BD PharMingen; 555824, 581, BD Biosciences), CD33 (551378, clone WM53, BD PharMingen; 565949, clone WM53, BD Horizon), CD15 (555401, clone HI98, BD Biosciences; IM1954U, clone 80H5, Beckman Coulter), Rabbit anti-human DRD1 antibody (324390, EMD Millipore) and Mouse anti-human DRD2 antibody (clone B-10, Santa Cruz). For secondary antibodies, anti-rabbit/mouse secondary antibodies (A31573, A31570, A31571; Thermo Fisher) were used. To minimize unspecific binding, samples were blocked with human FC block (eBioscience) and 5%–10% donkey serum (Jackson ImmunoResearch Laboratories). When appropriate, fluorescence minus one (FMO) controls and secondary antibody controls were used for optimized gating of target cell populations. For prospective purification experiments with DRD2, human AML cells were labeled with Rabbit anti-human DRD2 antibody (324393, EMD Millipore) and DRD2^{+} versus DRD2^{-} populations were purified for xenotransplantation and western blot analysis.

Across all experiments, 7-aminoactinomycin D (A07704, Beckman coulter) was used for live/dead cell discrimination. FACS sorting was performed using a FACSAria II sorter, and flow cytometry analysis was performed with a LSRII Cytometer (BD). FACSDiva (BD) software was used for data acquisition and FlowJo software (Tree Star) was used for analysis.

In vitro cell culture

Primary AML and healthy donor samples were cultured in StemSpan medium (StemCell Technologies, Inc.), supplemented with 100 ng/mL stem cell factor, 100 ng/mL Fms-related tyrosine kinase 3 ligand, and 20 ng/mL thrombopoietin (all sourced from R&D systems). Serum-free conditions were used during drug treatment or cAMP assays. Unless stated otherwise, a concentration of 10 μM for compounds, and 1:100 dilution was used for DRD1-Ab, and the data were compared to 0.1% DMSO or IgG control for *in vitro* treatment assays. Shandon Kwik-Diff stain (#9990700; Thermo Fisher) was used to visualize cultured cells by light microscopy.

Cyclic AMP measurement assay

For cAMP and CREB experiments, primary samples or AML cell lines were exposed to small molecules including (±)-SKF-38393 hydrochloride, R(+)-SCH-23390 hydrochloride, Thioridazine (all sourced from Sigma), Forskolin (Abcam), or antibody for 30 minutes in serum-free conditions, followed by cell lysis in HCL 1N. The supernatant containing cAMP was collected and applied to cAMP direct immunoassay kit (Millipore/Calbiochem) as per the manufacturer's instructions.

Methylcellulose colony forming unit assay and CFU imaging

The progenitor capacity of leukemic progenitors was evaluated by colony-forming unit (CFU) assays. Up to 50,000 AML MNCs, and 500-1000 CB/MPB Lin- cells were plated per well in semisolid methylcellulose media (StemCell Technologies, Inc.) following established protocols.[61] Progenitor capacity of human xenografts after exposure to TDZ *in vivo* was assayed after recovery of human cells from recipient mouse bone marrow, followed by seeding in methylcellulose media.[62,63] These analyses were performed using AML samples that gave rise to aberrant monocytic or granulocytic-enriched colonies that were confirmed to harbor patient-specific genetic aberrations when possible (Figures S1A and S1B). For whole-well CFU analysis, images were acquired at 2x using Operetta High Content Screening (Perkin Elmer) by means of epi-fluorescence illumination and standard filter sets. Image acquisition was performed using Harmony software (Perkin Elmer) and whole-well images were stitched in Columbus software (Perkin Elmer). A minimum of 40 cells was required for designation as a colony (Figure S1C).

Western blot analysis

Proteins were extracted in SDS Laemmli buffer, separated by SDS-PAGE, and transferred onto nitrocellulose membranes as previously described.[64] Membranes were blocked in PBS containing 5% skim milk and 0.1% TWEEN 20 (BioRad). For primary antibodies, rabbit anti-phospho-CREB (Ser133) (06-519, Millipore), mouse anti-CREB (9104, clone 86B10, Cell Signaling Technology), rabbit anti-DRD2 (AB5084P, Millipore) mouse anti-GAPDH (ab8245, clone 6C5, Abcam), mouse anti-Actin (MAB1501, clone C4, Millipore), and rabbit anti-Histone H3 (Cat#9715, Cell Signaling Technology) were used. Blot images were acquired using Chemidoc XRS system (Bio-Rad).

Measurement of dopamine levels in human plasma

Plasma was collected from the aqueous phase obtained during Ficoll separation of cells from human blood treated with anticoagulant and stored at -80°C. The oxidation status of the plasma was stabilized with 20 mL per 1mL of a solution containing ethylene glycol-bis (2-amino ethylether)-N,N,N',N'-tetraacetic acid (0.2M) and glutathione (0.2M) at pH = 7.5. The internal standard (3,4-Dihydroxybenzylamine) was added for further processing using solid phase extraction cartridges as per the manufacturer's recommendations (ChromSystems, Grafelfing, Germany). The samples were eluted into 120 mL and injected in triplicate within 24 hr in a High-Performance Liquid Chromatographic System (HPLC, Waters 2695) coupled to an Electro-Chemical Detector (Waters 2465). The HPLC system used an analytical reverse phase column (Atlantis dC18; 5 mm; 4.6x150mm; Waters) and an organic mobile phase (ChromSystems). The concentration of dopamine was calculated based on the area under the curve of the chromatograms with respect to the standards.

Chiral separation of TDZ enantiomers

Chiral separation protocols were developed by the Ontario Institute of Cancer Research (OICR). Briefly, TDZ racemic mixture was separated to positive and negative enantiomers using supercritical fluid chromatography with a chiral AD-H column. Free base enantiomers were recovered as viscous oils, converted to salt forms and subsequently purified as hydrochloride salt suitable for use in preclinical testing.

Analysis of AML-specific aberrations

Individual colonies were collected from methylcellulose after the progenitor CFU assay and genomic DNA was extracted using QIAamp DNA Micro Kit (QIAGEN) according to the manufacturer's instructions. To detect FLT3-ITD, 1:20 of the extracted genomic DNA was used for a PCR reaction using Platinum SuperFi DNA Polymerase (Thermo Fisher Scientific) and 0.5 mol l^{-1} each of primers targeting exons 13-15 of FLT3 gene (5′- attgtcgttttaaccctgctaat $-3'$ and 5- ttttgctaattccataagctgtt $-3'$). PCR was performed with denaturing at 98°C for 30 s, 40 cycles of denaturing, annealing, extension (98°C for 7 s,53°C for 10 s,72°C for 18 s), followed by final extension at 72°C for 5 minutes. The PCR product was visualized using ChemiDoc imaging system (BIO-RAD) after electrophoresis in a 1.5% agarose gel.

Deletion 7 was measured by droplet digital PCR (ddPCR) with 1:4 of the genomic DNA purified from individual colonies and subjected to ddPCR using BIO-RAD QX200 System. The result was analyzed using QuantaSoft software (BIO-RAD) The CNVs were calculated as the fraction of positive droplets containing a target divided by positive droplets containing a RPPH1 control locus. The TaqMan probes and primers used for the ddPCR (and their targeting gene and cytoband) are: Hs04327806_cn (targeting AUTS2 on 7q11.22) and Hs05538458_cn (as a control, targeting RPPH1 on 21q21.3).

Small mammal evaluation of QTc

QTc analysis was performed by Eurofins Scientific. Male Dunkin-Hartley guinea pigs weighing 350-450 g were anesthetized. Tracheotomy was performed and the animals were mechanically ventilated on animal placed on a heating pad, with circulating water at a temperature of 37-39C. The jugular vein and the carotid artery were cannulated for drug administration and blood pressure monitoring, respectively. ECG pin electrodes were positioned for the standard limb lead (Lead II). The carotid arterial catheter was connected to a pressure transducer and ECG cable signals were relayed to a Gould physiograph with outputs to a data acquisition system (Ponemah). The blood pressure and ECG were sampled at rates of 250 and 1000 hrtz, respectively. The heart rate values were obtained from the ECG. The animals were allowed to stabilize for a 30-minute period after instrumental prior to data collection. Vehicle, TDZ and enantiomers and Satol were injected via pre-cannulated jugular vein. QTc (Bazzett's), blood pressure and heart rate were recorded at minute 0, 1, 3, 5, 10, 20 and 30 after drug administration. A $\geq$ 5% prolongation of the QTc interval was considered a significant response.[46] Repeated-measures ANOVA followed by Dunnett's test was applied for statistical comparison.

RNA purification, PCR and Affymetrix

In xenograft assays, RNA was isolated from purified human leukemic xenografts (hCD45+CD33+) using a total RNA purification kit (Norgen biotek, ON, Canada) according to the manufacturer's instructions. RNA integrity was evaluated by 2100 Bioanalyzer (Agilent Technologies) and hybridized to Affymetrix Gene Chip Human Gene 2.0 ST arrays (London Regional Genomics Centre, ON, Canada). Output data was normalized using the Robust Multichip Averaging (RMA) algorithm with Partek Genomics Suite 6.6 software (Partek Inc.). Gene set enrichment analysis (GSEA) was carried out on normalized expression values of gene symbols using GSEA software v2.1.0 (Broad Institute). Curated gene set (C2) and Gene Ontology (GO, C5) gene set collections from Molecular Signatures Database (MSigDB) were used for GSEA analyses.

QUANTIFICATION AND STATISTICAL ANALYSIS

DRD gene expression in public datasets

Paired-end reads of the whole transcriptome RNA-sequencing data from TCGA and GTEx projects were retrieved after re-alignment of raw reads, quality control and batch effect correction.[47] Comparisons for healthy versus cancer transcript levels were available for a subset of tissues.[47] Thyroid samples include TCGA-tumor versus TCGA-normal plus GTEX. Kidney samples include TCGA-tumor kidney cortex (chromophobe renal cell carcinoma/kich[47]) versus TCGA-normal and GTEX. Colon samples include TCGA-tumor colon (adenocarcinoma/coad[47]) versus TCGA-normal and GTEX colon. Lung samples include TCGA-tumor for lung (squamous cell carcinoma/lusc[47]) versus TCGA-normal and GTEX.

Survival annotation based on TCGA gene expression data were available from the pathology atlas of Human Protein Atlas database.[65] Briefly, FPKM values were used to classify patients into "low" and "high" expression groups based on expression cut-off.[65] The prognosis of the two groups of patients was examined by Kaplan-Meier survival analysis, and survival outcomes were compared by log-rank tests.

Statistics

Data are described as mean ± standard error of the mean (SEM). Significant differences between groups were determined via unpaired two-tailed Student's t test, two-way analysis of variance (ANOVA) or linear regressions. If data failed to meet parametric requirements, $\log_{10}$ or square root transformation was applied prior to statistical evaluation, or non-parametric Mann–WhitneyU test was used. Prism (version 5.0a; GraphPad) software was used for all statistical analyses, and the criterion for statistical significance was $p \leq 0.05$. In Figure 1F, a data point corresponding to one mouse within the TDZ treated group was considered a significant outlier by Grubb's test ($p < 0.01$ two-sided, $Z = 2.038$) and this data point was removed.

Cell Reports Medicine, Volume 2

Supplemental Information

Abnormal dopamine receptor signaling allows selective therapeutic targeting of neoplastic progenitors in AML patients

Lili Aslostovar, Allison L. Boyd, Yannick D. Benoit, Justin Di Lu, Juan Luis Garcia Rodriguez, Mio Nakanishi, Deanna P. Porras, Jennifer C. Reid, Ryan R. Mitchell, Brian Leber, Anargyros Xenocostas, Ronan Foley, and Mickie Bhatia

Supplemental Figure 1

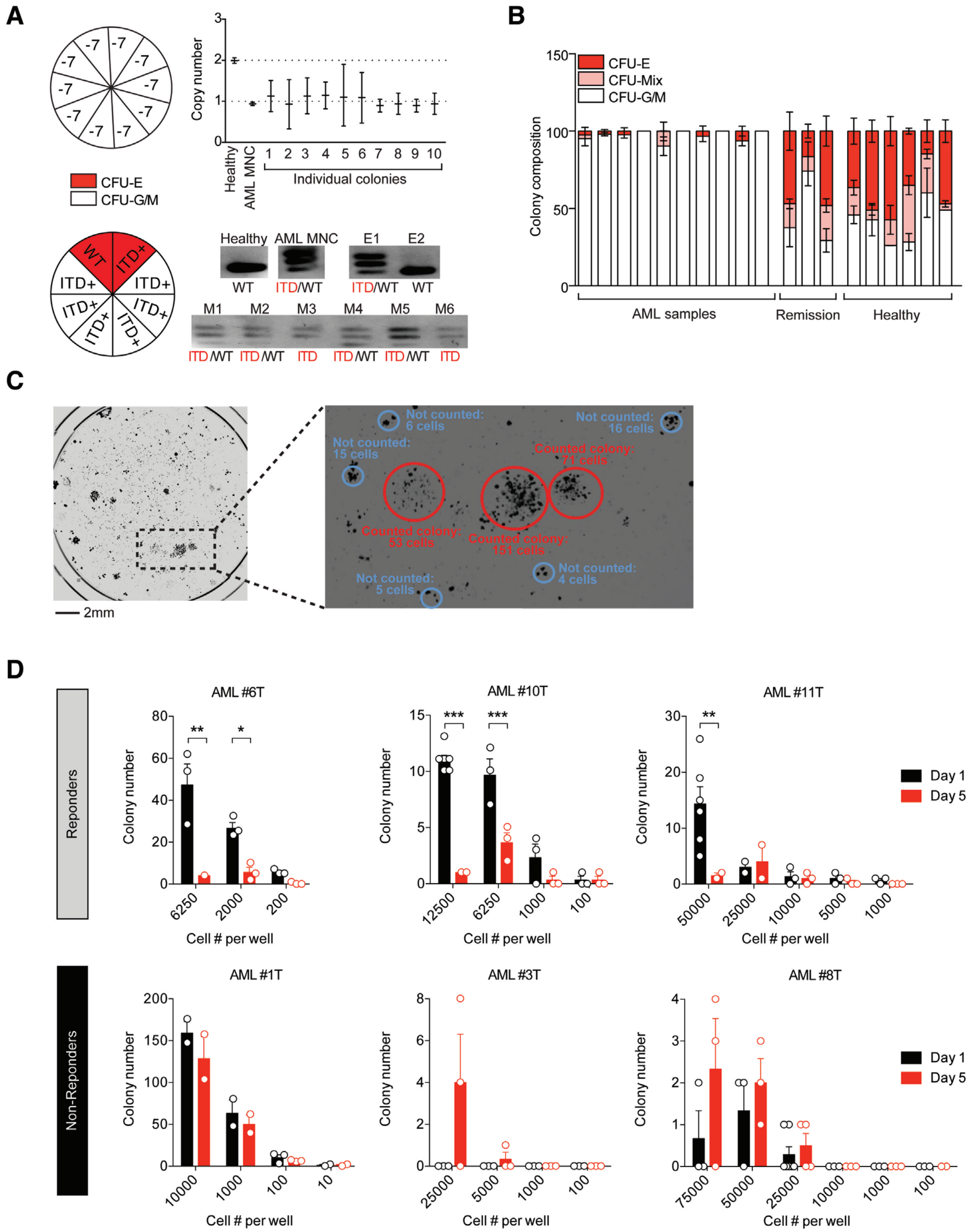

Figure S1. Characterization of AML patient samples for progenitor assays. Related to Figure 1. (A) Quantification of AML-related aberrations in granulocytic/monocytic ("G/M") versus erythroid ("E") colonies from two independent AML patients. Chromosome 7 deletion ("-7") was analyzed using droplet digital PCR system in 10 independent colonies by copy number variation analysis. Error bars indicate Poisson-based 95% confidence interval. FLT3-ITD mutation was tested using qRT-PCR analysis. Patient MNCs were used as a positive control and healthy donor samples were used as a negative control. (B) Based on Figure S1A, AML patient samples with over 90% enrichment for "G/M" colonies were prioritized for progenitor analyses, in contrast to diverse colony subtypes derived from healthy donor samples including CB and MPB and 3 remission cases of AML. (C) A representative whole well image shows contrast-adjusted calcein fluorescence of CFU colonies. The higher magnification image provides an example of our criteria used to score individual colonies for all experiments, based on a minimum requirement of 40 cells. Red circles indicate colonies that were counted, while blue circles indicate clusters that were not counted. (D) Candidate trial patient samples from either response group were interrogated for progenitor content at baseline (Day 1) and after clinical exposure to TDZ (Day 5) using limiting dilution analysis (LDA). *p<0.05, **p<0.01, ***p<0.001 (two-way ANOVA).

Supplemental Figure 2

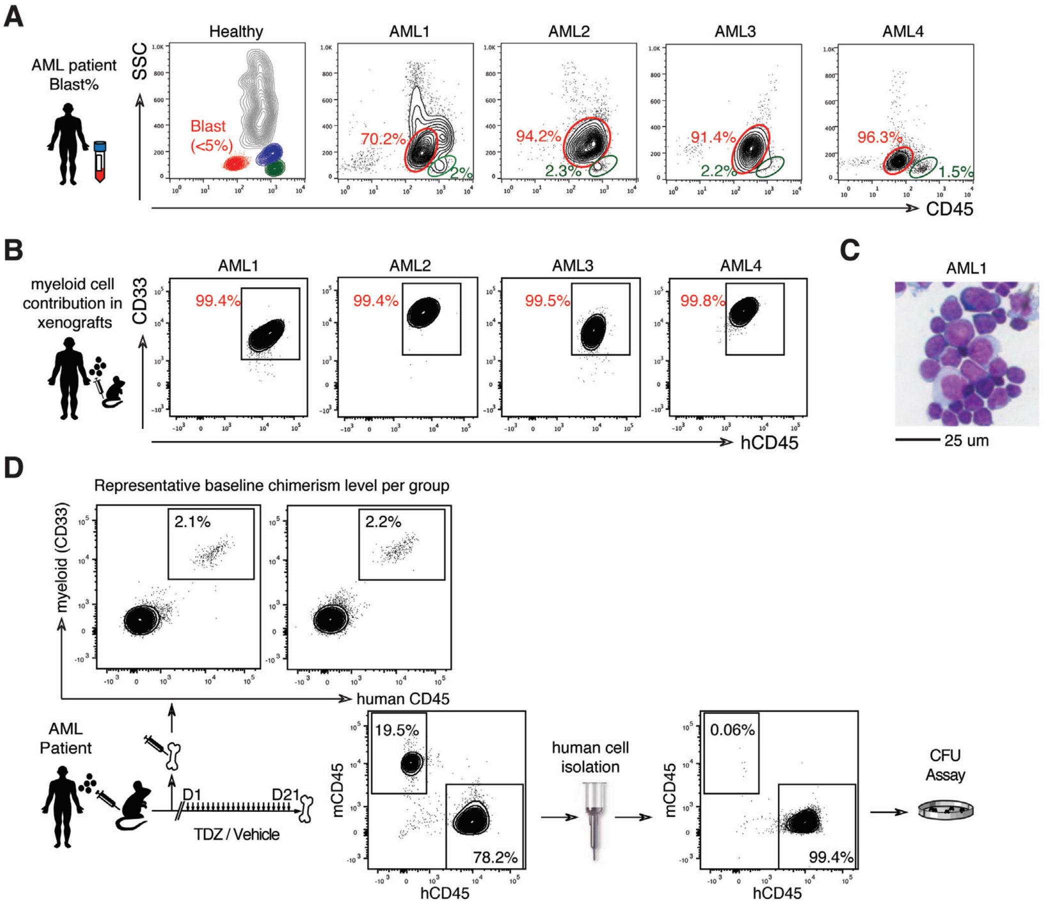

Figure S2. Characterization of AML patient samples for xenograft assays. Related to Figure 1. (A) FACS plots illustrate % leukemic blast (red gate) in AML patient samples. Green, blue and grey gates represent lymphocyte, monocyte and granulocytes, respectively. (B) AMLs 1-4 generate exclusively myeloid grafts in vivo. (C) Cytospin preparations showing blast morphology among human AML cells isolated from xenograft BM (AML 1). Original image capture had limited resolution and highest resolution is shown. (D) Recipient mice were assigned to TDZ versus vehicle control groups based on chimerism levels as quantified by BM aspirate sampling at approximately 3 weeks post-transplant, prior to the start of TDZ treatment. Representative FACS plot depicts the purity of isolated human leukemia cells prior to analysis of progenitor activity at the experimental endpoint.

Supplemental Figure 3

A

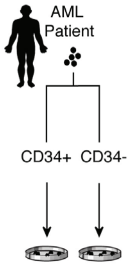

	CD34+		CD34-			
	Cell dose 1	CFU⁺ wells from total tested	Cell dose 1	CFU⁺ wells from total tested	Cell dose 2	CFU⁺ wells from total tested
AML 1	25k	3 of 4	5k	0 of 4	25k	0 of 4
AML 2	9k	3 of 3	9k	0 of 4	25k	0 of 4
AML 3	30k	4 of 5	30k	0 of 2		
AML 5	15k	1 of 4	15k	0 of 4		
AML 6	5k	3 of 3	5k	0 of 3	25k	0 of 3
AML 7	5k	3 of 3	5k	0 of 4		
AML 9	20k	1 of 4	20k	4 of 4		
AML 10	25k	4 of 4	25k	0 of 4		

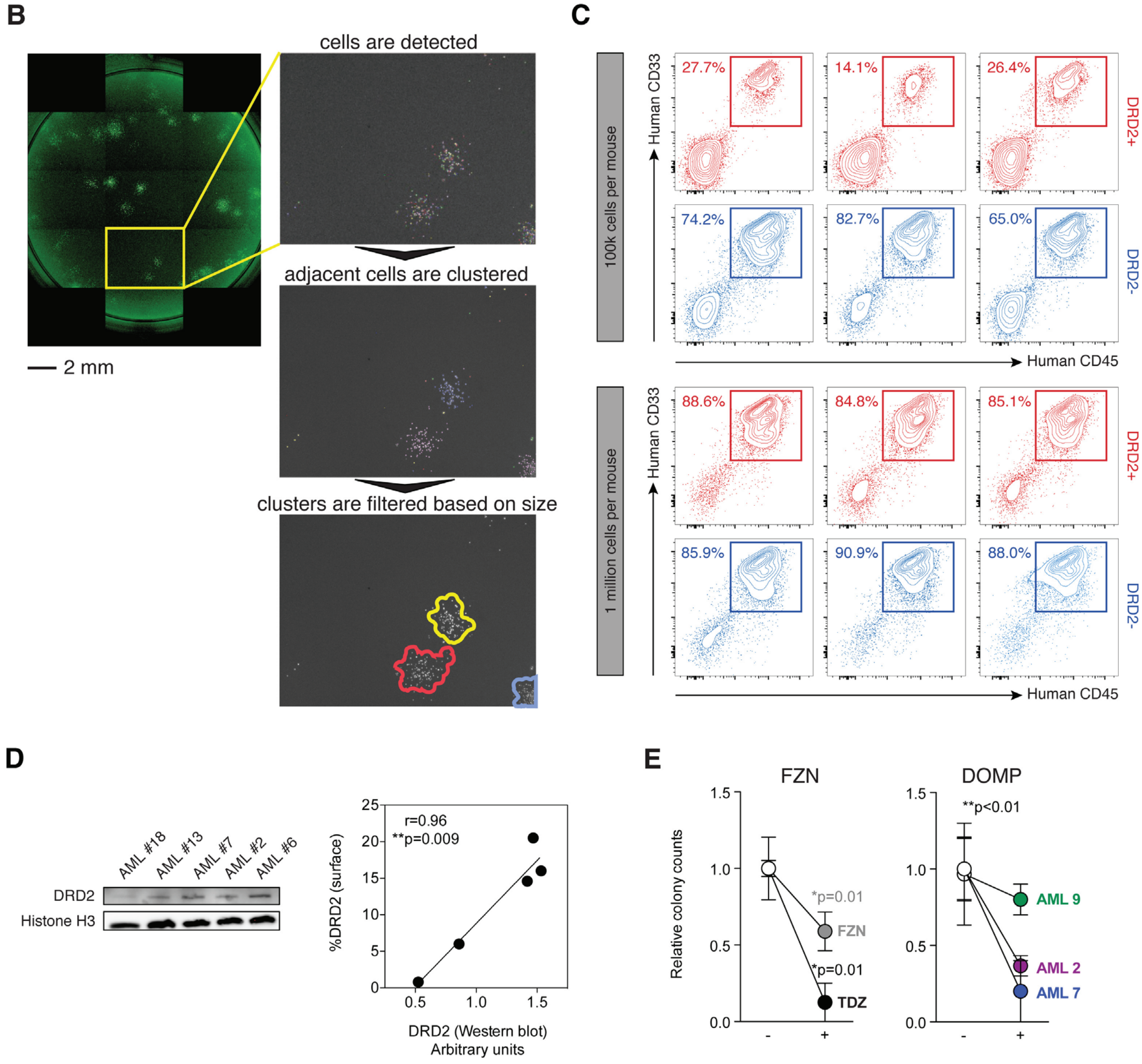

Figure S3. DRD2 signaling is functionally relevant to AML disease. Related to Figure 3. (A) CD34+ and CD34- subsets were FACS purified from AML patient MNCs for quantification of progenitor frequencies at various cells doses. (B) Identification of individual clusters and enumeration of cells within clusters as a measure of progenitor proliferative capacity using custom scripts. Original image capture had limited resolution and highest resolution is shown. (C) Human leukemic chimerism in mice transplanted with DRD2+ or DRD2- human AML cells. Note that plots from two of the mice at the 1 million cell dose are also shown in Figure 3E. (D) Western blot of DRD2 and Histone H3 (loading control) in 5 different primary patient samples (left). Correlation plot of cell surface DRD2 protein levels measured by flow cytometry vs. total DRD2 protein levels measured by Western blot. *p=0.009 (Pearson's correlation). Note that Western blot for patient AML 9 is also shown in Figure 3F. (E) Comparison of leukemic progenitor suppression after treatment with TDZ, fluphenazine hydrochloride ("FZN") and domperidone ("Domp") versus DMSO ("-"). FZN and TDZ treatments were performed using cells from patient AML 9. $n \geq 3$ CFU wells per condition. *p=0.01 (unpaired t-test), **p<0.01 (two-way ANOVA).

Supplemental Figure 4

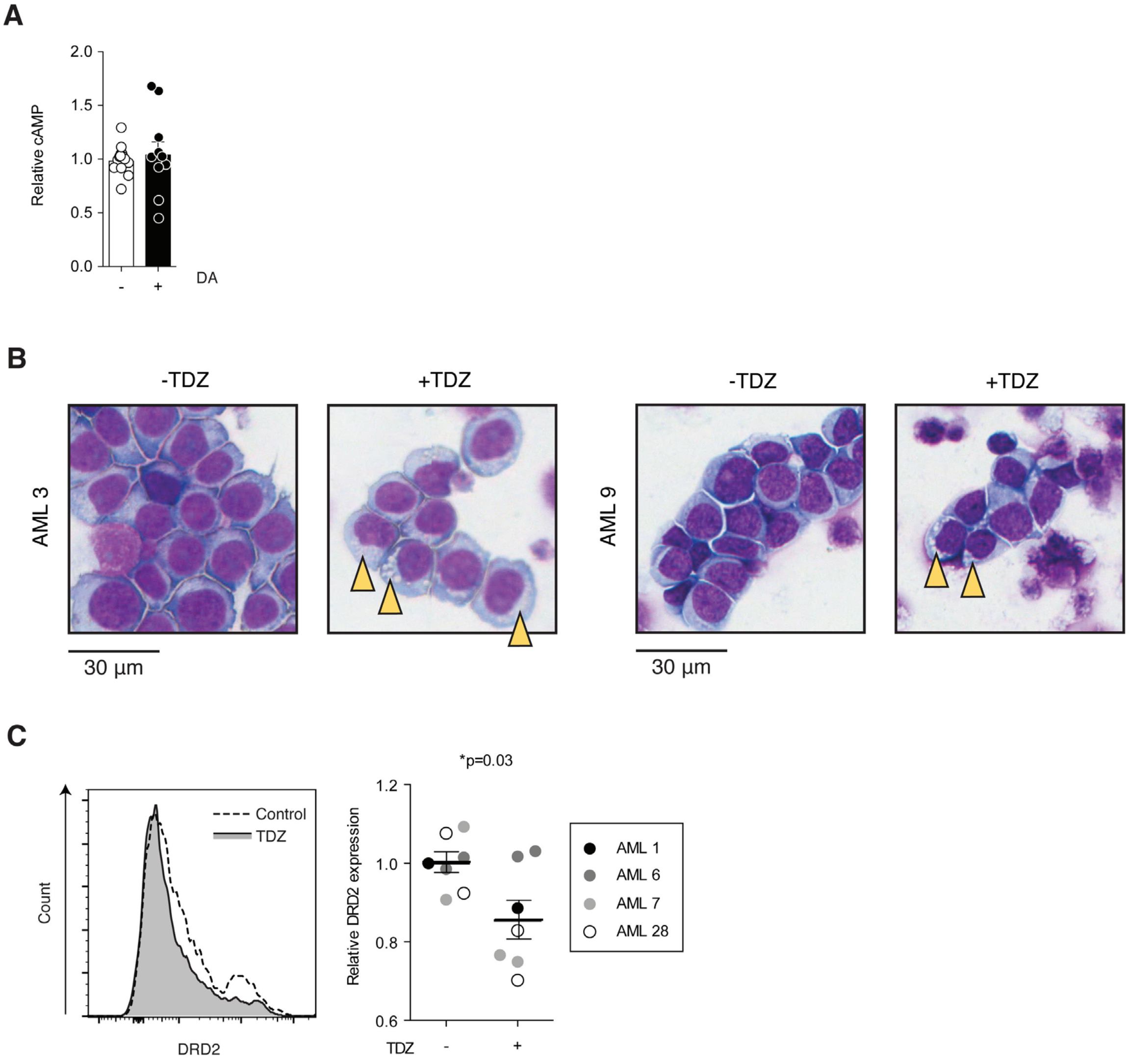

Figure S4. DRD2 signaling promotes cellular maturation and loss of self-renewal. Related to Figure 4. (A) cAMP levels in response to DA (100 nM) versus DMSO control, in AML cell line OCI-AML3. Data represent normalized values from *N*=5 independent experiments. (B) Cytospin preparations of AML after exposure to TDZ or vehicle control (DMSO). Yellow arrowheads indicate evidence of hematopoietic maturation (increased cytoplasmic vacuolization). Original image capture had limited resolution and highest resolution is shown. (C) FACS plot showing expression of DRD2 after in vitro exposure to TDZ or DMSO control (left). DRD2 frequencies were quantified for AMLs 1, 6, 7, and 28 (right). *p=0.03 (unpaired t-test).

Supplemental Figure 5

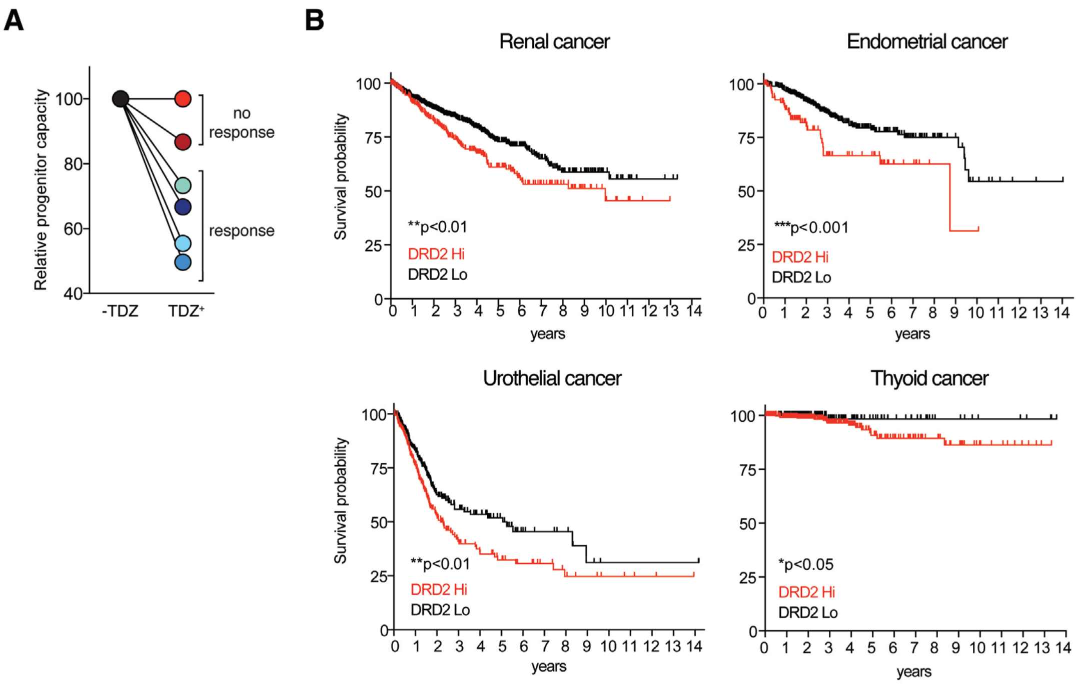

Figure S5. DRD2 is aberrantly expressed in tumors beyond AML. Related to Figure 5. (A) Trial patient samples obtained at baseline were exposed to TDZ+ versus DMSO control ("-TDZ"), followed by analysis of progenitor capacity. Patients included #1T, #8T for non-responders, and #2T, #6T, #10T, and #11T for responders. Data are normalized to DMSO control. (B) Survival analysis in cancer patients expressing high levels of DRD2 "Hi" versus low "Lo". Kaplan-Meier curves were generated using TCGA RNA-seq data with survival annotations provided by the pathology atlas. Log-rank test was used for statistical evaluation.

Table S1. Clinical details of AML patient samples. Related to Figure 1.

AML Sample ID	Disease stage	Cytogenetics	Molecular	Risk group*	%DRD2 in CD34+	%DRD1 in CD34+
1	Diagnosis	Normal	FLT3-ITD+, NPM1-	Intermediate-I	3.7	26.5
2	Diagnosis	NA	NA	NA	16.8	NA
3	Diagnosis	45, XX, del(5)(q22q33), -7[4]	NA	Adverse	NA	NA
4	Diagnosis	NA	NA	NA	NA	NA
5	Diagnosis	46, XY, 38 double minutes	NA	Intermediate-II	3.2	6.99
6	Relapse	45, XY, inv(3)(q21q26.2), -7[25]	NA	Adverse	8.1	5.1
7	Diagnosis	Normal	FLT3-ITD+, NPM1+	Intermediate-I	11.8	10.5
8	Diagnosis	Normal	FLT3-ITD+, NPM1-	Intermediate-I	2.2	15.2
9	Diagnosis	Normal	FLT3-ITD-, NPM1+	Favorable	1.3	26.5
10	Diagnosis	Normal	CBFß/MYH11+	Favorable	2.2	8.4
11	Diagnosis	Normal	FLT3-ITD+, NPM1-	Intermediate-I	3.2	NA
12	Diagnosis	Normal	NA	NA	0	1.2
13	Diagnosis	Normal	NA	NA	0.1	NA
14	Diagnosis	46,XX,inv(16)(p13q22)[25]	NA	Favorable	1.5	NA
15	Diagnosis	46,XX,add(4)(q31),t(16;16)(p13;q22)[25]	NA	Favorable	0.7	NA
16	Diagnosis	NA	NA	NA	1.4	NA
17	Diagnosis	47,XY,+13[24]/46,XY[1]	NA	Intermediate-II	0.8	NA
18	Diagnosis	46,XX,inv(16)(p13q22)[4]/ 47,idem,+22[21]	NA	Favorable	0	NA
19	Diagnosis	46,XX,t(8;21)(q22;q22)[25]	NA	Favorable	0.5	NA
20	Diagnosis	Normal	NA	NA	4.4	NA
21	Relapse	Normal	FLT3-ITD+, NPM1+	Intermediate-I	9.8	26.9
22	Diagnosis	NA	NA	NA	2.6	NA
23	Diagnosis	Normal	FLT3-ITD+, NPM1+	Intermediate-I	0.8	9.1
24	Diagnosis	Normal	FLT3-ITD+, NPM1-	Intermediate-I	7.7	NA
25	Diagnosis	46~47, XY, +9[3]/46, XY, del(15)(q11.2q15)[3]/46, XY, del(16)(q22)[2]/46, XY[6]	NA	Adverse	5.6	12.4
26	Relapse	Normal	FLT3-ITD+, NPM1+	Intermediate-I	7.5	NA
27	Relapse	42~46,idem,del(3)(p22p24), der(3)inv(3)(p21q21) del(3)(q21),del(7)(q32),add(18)(q21), add(20)(p12)	NA	Adverse	NA	NA
28	Diagnosis	NA	NA	NA	NA	NA

*European LeukemiaNet (ELN) criteria (Dohner et al., 2010)

Table S2. Gene Set Enrichment Analysis of upregulated pathways in control- vs. TDZ- exposed AML cells. Related to Figure 1.

Tissue	NAME	# of genes	NES	FDR q-val
Breast	SOTIRIOU_BREAST_CANCER_GRADE_1_VS_3_UP	138	-2.39	0.0000
	GINESTIER_BREAST_CANCER_ZNF217_AMPLIFIED_UP	65	-2.33	0.0000
	BERTUCCI_MEDULLARY_VS_DUCTAL_BREAST_CANCER_UP	188	-1.96	0.0019
	FARMER_BREAST_CANCER_CLUSTER_6	16	-1.92	0.0028
	JAZAERI_BREAST_CANCER_BRCA1_VS_BRCA2_UP	46	-1.90	0.0034
	POOLA_INVASIVE_BREAST_CANCER_UP	266	-1.87	0.0044
Lung	LOCKWOOD_AMPLIFIED_IN_LUNG_CANCER	198	-2.24	0.0000
	SHEDDEN_LUNG_CANCER_POOR_SURVIVAL_A6	425	-2.22	0.0001
	SWEET_LUNG_CANCER_KRAS_UP	456	-2.05	0.0007
	STEARMAN_LUNG_CANCER_EARLY_VS_LATE_UP	110	-1.91	0.0031
	KEGG_NON_SMALL_CELL_LUNG_CANCER	52	-1.66	0.0210
Blood	ROSS_LEUKEMIA_WITH_MLL_FUSIONS	68	-2.46	0.0000
	CASORELLI_ACUTE_PROMYELOCYTIC_LEUKEMIA_UP	162	-2.62	0.0000
	KEGG_CHRONIC_MYELOID_LEUKEMIA	69	-1.98	0.0016
	KEGG_ACUTE_MYELOID_LEUKEMIA	54	-1.93	0.0026
	PEPPER_CHRONIC_LYMPHOCYTIC_LEUKEMIA_UP	32	-1.81	0.0070
Liver	BOYAULT_LIVER_CANCER_SUBCLASS_G3_UP	178	-2.32	0.0000
	HOSHIDA_LIVER_CANCER_SUBCLASS_S1	220	-2.16	0.0002
	SAKAI_CHRONIC_HEPATITIS_VS_LIVER_CANCER_UP	76	-2.33	0.0000
Pancreatic, gastric and colon	GRUETZMANN_PANCREATIC_CANCER_UP	339	-1.94	0.0023
	KEGG_PANCREATIC_CANCER	67	-1.61	0.0295
	VECCHI_GASTRIC_CANCER_EARLY_UP	379	-1.91	0.0031
	AUNG_GASTRIC_CANCER	49	-1.65	0.0223
	GRADE_COLON_AND_RECTAL_CANCER_UP	258	-2.09	0.0005
Bladder	OSMAN_BLADDER_CANCER_UP	378	-2.91	0.0000
	LINDGREN_BLADDER_CANCER_CLUSTER_3_UP	305	-2.20	0.0001
Cervical	ROSTY_CERVICAL_CANCER_PROLIFERATION_CLUSTER	130	-2.51	0.0000
	OUELLET_OVARIAN_CANCER_INVASIVE_VS_LMP_UP	112	-2.38	0.0000
Other	WONG_EMBRYONIC_STEM_CELL_CORE	307	-2.17	0.0002
	WHITEFORD_PEDIATRIC_CANCER_MARKERS	107	-2.11	0.0003
	RAMALHO_STEMNESS_UP	193	-2.10	0.0004
	LIU_COMMON_CANCER_GENES	68	-1.77	0.0095
	LUI_THYROID_CANCER_CLUSTER_3	23	-1.66	0.0212
	KEGG_PROSTATE_CANCER	83	-1.52	0.0510

Table S3. KEGG acute myeloid leukemia gene list. Related to Figure 1.

Gene name	Gene description	Gene category/ family
AKT1	AKT Serine/Threonine Kinase 1	Protein coding/ Oncogene/ Protein kinase
AKT2	AKT Serine/Threonine Kinase 2	Protein coding/ Oncogene/ Protein kinase
AKT3	AKT Serine/Threonine Kinase 3	Protein coding/ Protein kinase
ARAF	A-Raf Proto-Oncogene, Serine/Threonine Kinase	Protein coding/ Protein kinase
BAD	BCL2 Associated Agonist Of Cell Death	Protein coding
BRAF	B-Raf Proto-Oncogene, Serine/Threonine Kinase	Protein coding/ Oncogene/ Protein kinase
CCNA1	Cyclin A1	Protein coding
CCND1	Cyclin D1	Protein coding/ Oncogene
CEBPA	CCAAT Enhancer Binding Protein Alpha	Protein coding/ Transcription factor/ Oncogene
CHUK	Component Of Inhibitor Of Nuclear Factor Kappa B Kinase Complex	Protein coding/ Protein kinase
EIF4EBP1	Eukaryotic Translation Initiation Factor 4E Binding Protein 1	Protein coding
FLT3	Fms Related Tyrosine Kinase 3	Protein coding/ Oncogene/ Protein kinase
GRB2	Growth Factor Receptor Bound Protein 2	Protein coding
HRAS	HRas Proto-Oncogene, GTPase	Protein coding/ Oncogene
IKBKB	Inhibitor Of Nuclear Factor Kappa B Kinase Subunit Beta	Protein coding/ Protein kinase
IKBKG	Inhibitor Of Nuclear Factor Kappa B Kinase Regulatory Subunit Gamma	Protein coding
JUP	Junction Plakoglobin	Protein coding
KIT	KIT Proto-Oncogene, Receptor Tyrosine Kinase	Protein coding/ Oncogene/ Protein kinase
KRAS	KRAS Proto-Oncogene, GTPase	Protein coding/ Oncogene
LEF1	Lymphoid Enhancer Binding Factor 1	Protein coding/ Transcription factor
LOC652346		
LOC652671		
LOC652799		
MAP2K1	Mitogen-Activated Protein Kinase Kinase 1	Protein coding/ Protein kinase
MAP2K2	Mitogen-Activated Protein Kinase Kinase 2	Protein coding/ Protein kinase
MAPK1	Mitogen-Activated Protein Kinase 1	Protein coding/ Protein kinase
MAPK3	Mitogen-Activated Protein Kinase 3	Protein coding/ Protein kinase
MTOR	Mechanistic Target Of Rapamycin Kinase	Protein coding/ Protein kinase
MYC	MYC Proto-Oncogene, BHLH Transcription Factor	Protein coding/ Transcription factor/ Oncogene
NFKB1	Nuclear Factor Kappa B Subunit 1	Protein coding/ Transcription factor
NRAS	NRAS Proto-Oncogene, GTPase	Protein coding/ Oncogene
PIK3CA	Phosphatidylinositol-4,5-Bisphosphate 3-Kinase Catalytic Subunit Alpha	Protein coding/ Oncogene
PIK3CB	Phosphatidylinositol-4,5-Bisphosphate 3-Kinase Catalytic Subunit Beta	Protein coding
PIK3CD	Phosphatidylinositol-4,5-Bisphosphate 3-Kinase Catalytic Subunit Delta	Protein coding
PIK3CG	Phosphatidylinositol-4,5-Bisphosphate 3-Kinase Catalytic Subunit Gamma	Protein coding
PIK3R1	Phosphoinositide-3-Kinase Regulatory Subunit 1	Protein coding/ Tumor suppressor
PIK3R2	Phosphoinositide-3-Kinase Regulatory Subunit 2	Protein coding
PIK3R3	Phosphoinositide-3-Kinase Regulatory Subunit 3	Protein coding
PIK3R5	Phosphoinositide-3-Kinase Regulatory Subunit 5	Protein coding
PIM1	Pim-1 Proto-Oncogene, Serine/Threonine Kinase	Protein coding/ Oncogene/ Protein kinase
PIM2	Pim-2 Proto-Oncogene, Serine/Threonine Kinase	Protein coding/ Protein kinase

Gene name	Gene description	Gene category/ family
PML	Promyelocytic Leukemia	Protein coding/ Transcription factor/ Oncogene
PPARD	Peroxisome Proliferator Activated Receptor Delta	Protein coding/ Transcription factor
RAF1	Raf-1 Proto-Oncogene, Serine/Threonine Kinase	Protein coding/ Oncogene/ Protein kinase
RARA	Retinoic Acid Receptor Alpha	Protein coding/ Transcription factor/ Oncogene
RELA	RELA Proto-Oncogene, NF-KB Subunit	Protein coding/ Transcription factor
RPS6KB1	Ribosomal Protein S6 Kinase B1	Protein coding/ Protein kinase
RPS6KB2	Ribosomal Protein S6 Kinase B2	Protein coding/ Protein kinase
RUNX1	Runt Related Transcription Factor 1	Protein coding/ Transcription factor/ Oncogene
RUNX1T1	RUNX1 Translocation Partner 1	Protein coding/ Transcription factor/ Oncogene
SOS1	SOS Ras/Rac Guanine Nucleotide Exchange Factor 1	Protein coding
SOS2	SOS Ras/Rho Guanine Nucleotide Exchange Factor 2	Protein coding
SPI1	Spi-1 Proto-Oncogene	Protein coding/ Transcription factor
STAT3	Signal Transducer And Activator Of Transcription 3	Protein coding/ Transcription factor
STAT5A	Signal Transducer And Activator Of Transcription 5A	Protein coding/ Transcription factor
STAT5B	Signal Transducer And Activator Of Transcription 5B	Protein coding/ Transcription factor
TCF7	Transcription Factor 7	Protein coding/ Transcription factor
TCF7L1	Transcription Factor 7 Like 1	Protein coding/ Transcription factor
TCF7L2	Transcription Factor 7 Like 2	Protein coding/ Transcription factor
ZBTB16	Zinc Finger And BTB Domain Containing 16	Protein coding/ Transcription factor/ Oncogene

Table S4. Progenitor CFU assays after *in vivo* exposure to TDZ. Related to Figure 1.

Patient sample	Cell # seeded per well	Colony count DMSO	Colony count TDZ	Average colony count (DMSO)	Relative colony count DMSO	Relative colony count TDZ
AML #1	100k	79	49	74.7	1.06	0.66
		69	49		0.92	0.66
		76			1.02	
	75k	61	29	54.0	1.13	0.54
		61	34		1.13	0.63
		40			0.74	
AML #2	25k	39	3	26.5	1.47	0.11
		43	2		1.62	0.08
		24	0		0.91	0.00
		18			0.68	
		20			0.75	
		15			0.57	
AML #3	25k	6	3	6.3	0.95	0.47
		5	4		0.79	0.63
		8	4		1.26	0.63

Table S5. TDZ suppresses leukemic progenitors exclusively in DRD2+ AML. Related to Figure 3.

Patient sample	Group	Colony count DMSO	Colony count TDZ	Average colony count (DMSO)	Relative colony count DMSO	Relative colony count TDZ
AML #1	DRD2+	47	9	32.0	1.47	0.28
		37	17		1.16	0.53
		16	5		0.50	0.16
		28	18		0.88	0.56
AML #5	DRD2+	89	30	82.8	1.08	0.36
		99	38		1.20	0.46
		59	49		0.71	0.59
		84			1.02	
AML #6	DRD2+	22	13	18.8	1.17	0.69
		13	8		0.69	0.43
		22	13		1.17	0.69
		18	5		0.96	0.27
AML #7	DRD2+	10	0	7.3	1.38	0.00
		3	2		0.41	0.28
		8	3		1.10	0.41
		8	1		1.10	0.14
AML #8	DRD2+	21	14	21.0	1.00	0.67
		16	19		0.76	0.90
		26	14		1.24	0.67
AML #9	DRD2+	2	0	2.0	1.00	0.00
		2	0		1.00	0.00
		1	0		0.50	0.00
		3	1		1.50	0.50
AML #10*	DRD2+	3	1	3.4	0.88	0.29
		5	1		1.47	0.29
		4	3		1.18	0.88
		3	1		0.88	0.29
		2	1		0.59	0.29
		3	0	1.4	2.14	0.00
		0	1		0.00	0.71
		1	1		0.71	0.71
		2	0		1.43	0.00
		1	2		0.71	1.43
AML #11	DRD2+	1	0	1.8	0.57	0.00
		1	1		0.57	0.57
		3	0		1.71	0.00
		2	0		1.14	0.00
AML #12	DRD2lo	9	36	15.3	0.59	2.36
		30	21		1.97	1.38
		11	2		0.72	0.13
		11	9		0.72	0.59
AML #13	DRD2lo	222	181	194.7	1.14	0.93
		204	181		1.05	0.93
		158	131		0.81	0.67

Patient sample	Group	Colony count DMSO	Colony count TDZ	Average colony count (DMSO)	Relative colony count DMSO	Relative colony count TDZ
Cord blood #1	Healthy	44	33	33.5	1.31	0.99
		29	41		0.87	1.22
		32	35		0.96	1.04
		29	30		0.87	0.90
Cord blood #2	Healthy	52	52	56.3	0.92	0.92
		57	52		1.01	0.92
		63	43		1.12	0.76
		53	39		0.94	0.69
Cord blood #3	Healthy	45	37	46.0	0.98	0.80
		50	40		1.09	0.87
		46	33		1.00	0.72
		43			0.93	

*Two independent experiments were performed with cells from AML #10
